# A systematic review of the epidemiology of human monkeypox outbreaks and implications for outbreak strategy

**DOI:** 10.1371/journal.pntd.0007791

**Published:** 2019-10-16

**Authors:** Ellen M. Beer, V. Bhargavi Rao

**Affiliations:** 1 Department of Infectious Diseases Epidemiology, London School of Hygiene and Tropical Medicine, London, United Kingdom; 2 Manson Unit, Médecins sans Frontières (MSF) UK, London, United Kingdom; NIAID Integrated Research Facility, UNITED STATES

## Abstract

Monkeypox is a vesicular-pustular illness that carries a secondary attack rate in the order of 10% in contacts unvaccinated against smallpox. Case fatality rates range from 1 to 11%, but scarring and other sequelae are common in survivors. It continues to cause outbreaks in remote populations in Central and West Africa, in areas with poor access and weakened or disrupted surveillance capacity and information networks. Recent outbreaks in Nigeria (2017-18) and Cameroon (2018) have occurred where monkeypox has not been reported for over 20 years. This has prompted concerns over whether there have been changes in the biology and epidemiology of the disease that may in turn have implications for how outbreaks and cases should best be managed. A systematic review was carried out to examine reported data on human monkeypox outbreaks over time, and to identify if and how epidemiology has changed. Published and grey literature were critically analysed, and data extracted to inform recommendations on outbreak response, use of case definitions and public health advice. The level of detail, validity of data, geographical coverage and consistency of reporting varied considerably across the 71 monkeypox outbreak documents obtained. An increase in cases reported over time was supported by literature from the Democratic Republic of Congo (DRC). Data were insufficient to measure trends in secondary attack rates and case fatality rates. Phylogenetic analyses consistently identify two strains of the virus without evidence of emergence of a new strain. Understanding of monkeypox virulence with regard to clinical presentation by strain is minimal, with infrequent sample collection and laboratory analysis. A variety of clinical and surveillance case definitions are described in the literature: two definitions have been formally evaluated and showed high sensitivity but low specificity. These were specific to a Congo-Basin (CB) strain–affected area of the DRC where they were used. Evidence on use of antibiotics for prophylaxis against secondary cutaneous infection is anecdotal and limited. Current evidence suggests there has been an increase in total monkeypox cases reported by year in the DRC irrespective of advancements in the national Integrated Disease Surveillance and Response (IDSR) system. There has been a marked increase in number of individual monkeypox outbreak reports, from outside the DRC in between 2010 and 2018, particularly in the Central African Republic (CAR) although this does not necessarily indicate an increase in annual cases over time in these areas. The geographical pattern reported in the Nigeria outbreak suggests a possible new and widespread zoonotic reservoir requiring further investigation and research. With regards to outbreak response, increased attention is warranted for high-risk patient groups, and nosocomial transmission risks. The animal reservoir remains unknown and there is a dearth of literature informing case management and successful outbreak response strategies. Up-to-date complete, consistent and longer-term research is sorely needed to inform and guide evidence-based response and management of monkeypox outbreaks.

## Author summary

Monkeypox is a zoonotic infectious disease characterised by a pustular rash indistinguishable from smallpox, and systemic illness that can range from mild to fatal. Outbreaks predominantly affect remote populations in Central and West Africa. In 2017 and 2018, outbreaks were reported in Nigeria and Cameroon having been unreported for 20 years. We review monkeypox outbreak events occurring since 1970 to investigate if the pattern of outbreaks, person-to-person transmission and virus strain has changed and if so, whether this has implications for outbreak response strategies in low-resource settings. We found that recent literature continues to support an increase in reported outbreaks and number of cases by year in the Democratic Republic of Congo and number of outbreak reports per year in the Central African Republic. We highlight the importance of prioritising high-risk patient groups, remaining vigilant of nosocomial transmission and present that genetic strains remain unchanged. This study informs epidemiologists and outbreak response teams of the source and nature of the limited epidemiological data available on monkeypox outbreaks and may allow optimisation of public health advice and inform choice of suspected case definitions in field settings. Several recommendations are also made for further research efforts.

## Introduction

Between August 2017 and August 2018, human monkeypox outbreaks were reported in the Democratic Republic of Congo (DRC), Central African Republic (CAR), Cameroon, Republic of Congo (ROC), Liberia and Nigeria. Following the announcement of an outbreak in Nigeria in October 2017, an informal WHO meeting was called. The meeting report summarised that “*endemic monkeypox has been reported from more countries in the past decade than during the previous 40 years*”.[1]

A gradual increase between 1980 and 2013 in monkeypox case numbers has been suggested and supported in the literature[1, 2] but the extent to which recent outbreaks fit this trend is unclear. Moreover, the recent apparent increase monkeypox reports in areas after a hiatus raises questions about a change in the epidemiological pattern.

To our knowledge, a review of the relevant monkeypox literature to inform operational guidance in low-resource settings with limited access to diagnostics and therapeutics has not been carried out. This review aims to critically analyse the literature in particular addressing 3 issues:-

- Systematically review reported data on the epidemiology of monkeypox outbreaks
- Determine *if*, *how*, *and to what extent* the epidemiology of monkeypox outbreaks is changing and discuss the implications of findings for outbreak control strategies
- Systematically review ‘suspected case’ definitions used in outbreak response including rationale and implications for hospitalisation and transmission.

### Background

Human monkeypox was discovered in 1970 in an infant who had presented with smallpox-like eruptions in DRC.[3] The orthopoxvirus had first been isolated from skin lesions on an imported macaque in a Danish laboratory in 1958 and was known to cause outbreaks in captive primates.[4] Orthopoxvirus antibodies have been detected in multiple rodent and primate species in Central and West Africa but live monkeypox virus (MPXV) has only been isolated from sylvatic animals twice and the definitive reservoir remains unclear.[5–9]

Typically a 1-4 day febrile prodrome with headache and fatigue is followed by centrifugal development of deep well-circumscribed macular-papular, vesicular, pustular and finally crusted scab lesions.[10] Lesions last for around 1-3 days at each stage and progress simultaneously. Unlike smallpox, lymphadenopathy may develop before or during the rash.

The spectrum of disease ranges from mild to severe and fatal.[10] Recorded complications include vomiting and diarrhoea, conjunctivitis and corneal scarring, sepsis, encephalitis, and bronchopneumonia.[8] Permanent pitted scarring secondary to bacterial superinfection is a common long-term sequela.[8] Miscarriage and more severe disease have been reported in pregnant women.[11–13]

Smallpox vaccine has been estimated to confer on the order of 85% effectiveness against monkeypox. Residual immunity from past vaccination substantially reduces the frequency and intensity of clinical signs and symptoms.[10, 14] Reported case fatality rates (CFR) range from 0-11% in unvaccinated individuals.[10] Immunocompromised individuals, e.g. with untreated HIV infections, are vulnerable to more serious disease and higher risk of fatality.[15]

Monkeypox outbreaks typically occur in populations who hunt, kill, handle and consume bushmeat.[6, 7, 16] Evidence supports primary introduction via lesion material introduced percutaneously, mucocutaneously or via respiratory droplet.[17, 18]

Incubation period is around 7-14 days.[19] Patients are presumed infectious from rash onset until desquamation 4 weeks later.[8] Person-to person spread can occur via respiratory droplets, placenta, direct contact with skin abrasions or fomites.[19, 20]^,^[21]

Two main clades of human monkeypox virus (MPXV) have been identified: the West African (WA) strain and the Congo Basin (CB) strain. The latter has been associated with greater morbidity, mortality and human-to-human transmission.[22]

Virus isolation, electron microscopy and PCR are considered the gold standard techniques used to confirm MPXV infection.[23–25] However most cases in remote settings are clinically diagnosed. Chickenpox is frequently mistaken for monkeypox during monkeypox outbreaks.[26–28]

There are a select number of compounds developed for smallpox that could be tested widely for treatment efficacy with monkeypox.[29] Currently management of monkeypox cases in endemic areas is supportive. Use of vaccinia vaccine for prevention of transmission of monkeypox in at-risk individuals in endemic areas has had little focus since a fatality occurred after vaccinating an HIV-positive individual. Unknown HIV prevalence as well as difficulties accessing and safely delivering the vaccine means this method of prevention is rarely used in endemic settings.[15]

## Methods

**Fig 1:**
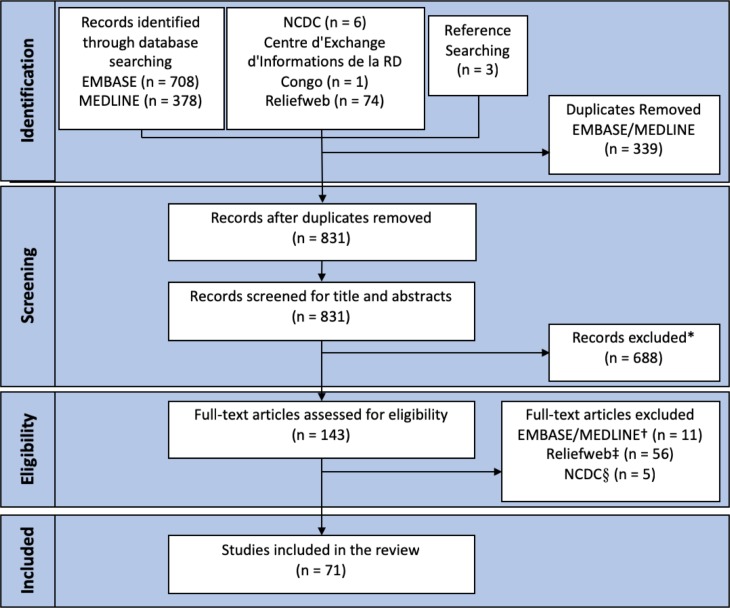
Study Selection. *287 were experimental laboratory or animal model studies, 196 were unrelated to monkeypox, 24 were animal serology studies, 29 pertained to non-clinical diagnostics, 15 were prevention studies, 39 were literature reviews, 40 concerned antivirals, 10 focussed on clinical characteristics, 12 were serology studies, 17 were author correspondence documents, 9 were projection models, 6 were varicella zoster virus (VZV) co-infection studies, 2 were regulation documents, 3 focussed on health workers †6 were inaccessible, 2 were documents that provided insufficient information after contacting authors, 2 were abstracts with insufficient information, 1 did not provide information on management after reading the full text ‡56 were the least up-to-date records §5 were the least up-to-date.

The Preferred Reporting Items for Systematic Reviews and Meta-Analyses (PRISMA) Statement checklist for systematic reviews was used as a reference protocol for this review.[30] Our search strategy to obtain published and unpublished literature is illustrated in Fig 1 (see S1 Text for search terms and S1 Table for inclusion/exclusion criteria). National public health or disease control departments of countries known ever to have reported monkeypox were searched. The keywords ‘monkeypox’ OR ‘monkeypox virus’ were used. All articles published prior to August 15^th^, 2018 in English were considered.

When multiple outbreak updates were found, the most up-to-date situation reports were selected, working backwards until no more new information was obtained. Reference chain searching was used, and inclusion/exclusion criteria applied. Given the aim to identify all literature pertaining to monkeypox outbreaks, and the high volume of grey literature, this should have enabled all possible papers to be screened.

Grey literature pertaining to the recent outbreak in Nigeria was requested from the director of the Nigerian Centre for Disease Control (NCDC) through personal communication. Individual authors of 4 conference abstracts[31–34] were contacted to obtain full-texts.

The following data were extracted from each document: authors, location, literature type, publication date, cohort/outbreak date, aim, methods, epidemiological characteristics (affected-population characteristics, number of cases, number of confirmed cases, number of fatalities, attack rate (primary, secondary, household or other), reproduction number, virus strain, vaccination status), risk factors for transmission, risk of bias; case definitions; case management with antibiotics. A data extraction table was piloted and refined. Once the table was complete, the data were moved to a Microsoft Excel spread sheet for analysis.

Epidemiological characteristics and risk factors were stratified by outbreak, location and year. Odds ratios (ORs) were calculated (where possible) and expressed with 95% confidence intervals (CI). Values used to calculate Secondary Attack Rates (SARs) and Case fatality rates (CFRs) were extracted and represented as proportions with 95% CI. SAR was defined as the proportion of individuals who encountered a confirmed or suspected case of monkeypox within a defined infectious period, who subsequently developed the disease within the accepted incubation period. A meta-analysis of SAR stratified by household vs. non-household contact and vaccination status was conducted using a random effects model in STATA. Outbreak characteristics such as gender, age and CFR were described using tables rather than meta-analysis techniques due to the heterogeneity in data collection methods and insufficient data to calculate CI in the case of median age.

Quality assessment tools were applied to evaluate the risk of bias rather than to exclude low quality literature. In the case of outbreak investigation and situation reports, risk of bias was manually described and critically analysed within this review rather than formally assessed and graded. Formal assessment was considered inappropriate for papers that do not follow a formal structure.

## Results

A total of 71 documents were included in the review (shown in Fig 1): 23 situation reports, 12 analyses of surveillance data cohorts, 11 outbreak investigation reports, 9 case reports, 8 descriptions of surveillance data, 3 press reports, 2 case-control studies, 1 cross-sectional study, 1 mixed case-control and cross-sectional study and 1 modelling study.

Publications summarising the 2017-18 Nigeria outbreak were not available at the date of correspondence with NCDC (14/08/2018).

All documents were published or made accessible between 13/10/1972 and 27/07/2018 and described monkeypox cases and outbreak data collected between October 1970 and July 2018.

**Fig 2:**
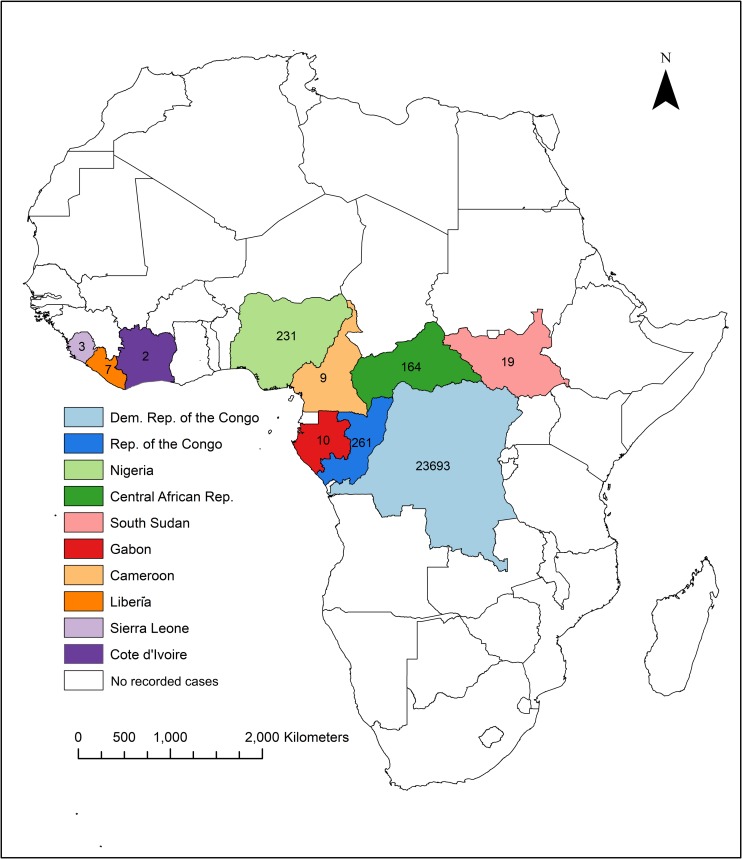
Map of countries and total number of suspected cases identified by this review, 1970-2018*. Created using ArcGIS. *Suspect cases in DRC are reported to exceed 1000 per year[1] and therefore the total reported cases is likely to exceed what was accessible for review.

Outbreak and surveillance data were identified from 10 countries across Central and West Africa: DRC/Zaire (n = 32), CAR (n = 7), ROC (n = 7), Nigeria (n = 5), Liberia (n = 4), Southern Sudan (now South Sudan) (n = 3), Cameroon (n = 2), Cote d’Ivoire (n = 1), Gabon (n = 1) and Sierra Leone (n = 2) (Fig 2). Eight documents pertained to the US outbreak. Three studies reported data from or compared data between 2–3 countries. Two additional studies analysed (genomic) data across multiple (>5) countries.

### Epidemiology

Most available age data came from the DRC, where the case burden was consistently concentrated in children. Over 80% of cases recorded in the 1970-9, 1981-6 and 1996-7 cohorts were <15 whilst less than 50% of the population were aged <15 during these times.[35] There has been a small steady increase in median age of cases since 1970 (see S2 Table). Outside the DRC age data were sparse and inconsistent. Earlier outbreaks were predominantly in children under-10 years, but otherwise higher median ages for smaller numbers of cases were seen. The median age of 228 suspected cases in the recent Nigeria outbreak was 30. Male cases were reported more frequently than female cases in 18 out of 26 reports in which gender information were available.

With few exceptions, outbreaks of monkeypox have consistently occurred in populations living in rural areas, in small villages (less than 1000 people) adjacent to or within humid evergreen tropical forests – at the so-called human-animal interface.[16, 36–52] Many outbreaks continue to occur in areas affected by armed conflict and mass population displacement. [6, 52–54] A single outbreak in a dry grassland area of Sudan in 2005 was attributed to movement of an infected individual from DRC.[49, 50, 55, 56] Infected imported rodents from Ghana caused an outbreak in families purchasing infected prairie dogs in the US in 2003. The characteristics of the population affected in the recent outbreak in Nigeria have not yet been described. Clusters of cases are stated to have ‘*no epidemiological linkages across states’* and the outbreak is suggested to reflect an underlying epizootic.[57]

Fifteen papers discussed virus strain in relation to human outbreaks.[6, 22, 37, 41, 49, 50, 52, 53, 55, 56, 58–63] Two papers conducted phylogenetic analyses of multiple isolates across different countries.[22, 60] A total of 40 human monkeypox samples collected between 1970 and 2010 were classified into 2 distinct clades. Isolates from the DRC, ROC, Gabon and Cameroon form the CB strain, and isolates from Nigeria, Cote d’Ivoire, Sierra Leone, Liberia and the USA (Ghana) form the WA clade.[22, 60, 63]

Twelve documents characterised strains from single outbreaks or multiple isolates from a cohort of cases in a single country. Three of 22 MPXV-positive samples from the 2017-18 Nigerian outbreak were 94-100% identical to a 1971 sample from Abia state.[58] A description of the remaining 19 isolates tested, reasons for the selection of 29 samples out of an available 56, and the results for 7 additional PCR reads were not provided.

Within the DRC, analysis of 23 samples from human cases suggest four lineages exist within the CB clade.[59] Each lineage was associated with a different outbreak locality, and different grade of severity. One deletion and resultant gene-loss was identified in 10 out of 16 samples in one lineage. Out of 8 available samples with this deletion, 7 were obtained from secondary cases (p = 0.05). Authors suggested this association with human-to-human transmission may reflect adaptations to human transmission in the MPXV genome.

#### Number of Outbreaks and incidence

**Fig 3:**
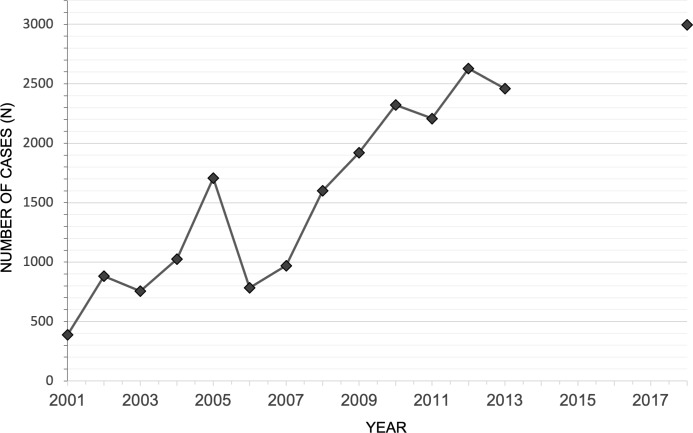
National Surveillance case burden in the DRC, by year, 2001-July 2018*. *ISDR data from the DRC were available from 2001-13, and 2018.[42, 64].

There has been an increase in number of individual outbreak reports over time since 1970. A total of 35 individual outbreaks have been reported outside the DRC, 20 of which occurred since 2010 (see S6 Table). Within the DRC, monkeypox reporting varies by period of surveillance. Intensified surveillance was carried out between the years 1970-79, 1981-86, and 2005-7 specifically in Sankuru District accounting for increased reports during these times.[15] Passive surveillance has been implemented since 2000 when monkeypox became reportable to Integrated Disease Surveillance and Response (ISDR). Available ISDR data shows a progressively increasing annual case burden since 2001 (Fig 3).

Two studies from the DRC support an increase in incidence of human monkeypox (mainly clinically diagnosed) since the 1970-1990 period. A 20-fold increase was reported between 1981-86 (0.72 per 10,000 population) and 2006–07 (14.42 per 10,000) when analysing active surveillance data from 9 health zones (HZ).[2] A rise between 2001 and 2013 was also reported; specifically a 45% increase between 2008 to 2013 from 21.3 cases per 10,000 population to 28.4 per 10,000.[64] Tetanus and acute flaccid paralysis (AFP) remained stable over this period, used by the author as a control for changes in IDSR reporting patterns and suggests that this per-unit population rise is unlikely to be due to population growth.

A modelling study estimated true national case burdens could be between 5 and 15 times the reported numbers although methods were not provided.[65] ISDR data were reported from just 27% of all HZs in 2013. Increases in number of cases per outbreak have been reported at HZ-level since 2013.[41, 66]

Authors reported an increase in case burden by outbreak in a study in CAR but longitudinal data were not available to support this.[54] An outbreak with 10 cases was reported on the ROC/DRC border where monkeypox had not been reported previously, following an NGO health education programme.[52]

#### Possible Animal-to-human transmission

Animals suspected to be responsible for monkeypox infections in the DRC between 1981 and 1986 were described extensively in 1988.[67] This review found 22 reports published between 1988 and 2018 where an animal source was identified (USA n = 5). Eight papers investigated but could not identify an animal source (4 case reports, 2 outbreak investigation reports, 2 situation reports). Twenty-seven papers did not report any animal source or other risk factors for an outbreak; 20 were situation reports, 3 were press reports, 2 were descriptions of surveillance data, one was a case report and one was an outbreak investigation report.

Consumption, hunting and handling were practices implicated in the primary contraction of monkeypox in 6 reports.[8, 41, 49, 53, 54] Studies identified after 1988 did not find any previously unknown species associated with monkeypox outbreaks in the DRC. Only squirrels (*Xerus erythropus*), Cane rats (*Thryonomis*), and Bemba (wild rodents) have been implicated in CAR.[49, 50, 54]

One case-control study and one conference abstract measured risk of animal-to-human transmission by animal-type and exposure-type (see S3 Table).[68, 69] Another case-control study compared behavioural risk factors.[40] Primary cases were reportedly more likely to sleep on the floor, less likely to eat duiker, prepare wild animal meat to cook or live in a house that had a door than healthy household controls in the Bokungu outbreak, DRC (n = 15 primary cases, n = 50 matched community controls).[40] No significant risk was associated with having animals in the house, finding dead animals around the house, coming into contact with animal excrement, invasive contact with an animal, or hunting or cooking wild animals. The authors postulated that young males seemed implicated in transmission to new households and sharing utensils could lead to inter-human transmission, but no evidence was provided for this.

#### Human-to-human transmission

**Fig 4:**
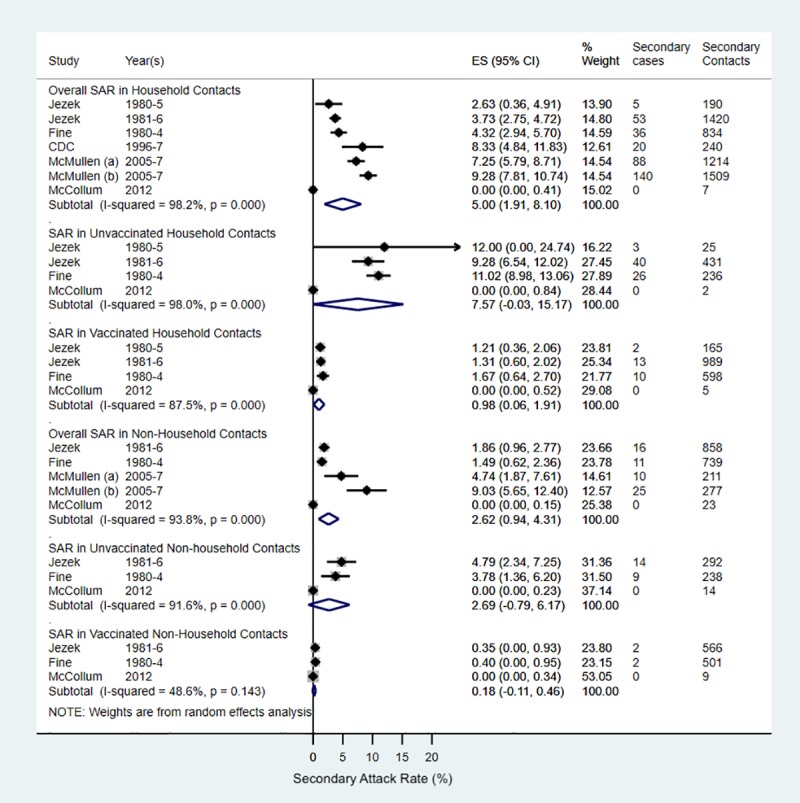
Forest plot of SARs in the DRC (CB strain) stratified by household contacts, and contact vaccination status. *Created using STATA*. *Diamonds represent summary SAR*. *P values following the I*^*2*^ *statistics represent the χ*^*2*^ *test for heterogeneity*. *SAR were calculated as number* of secondary cases/number of contacts (see S5 Table). Weights are from random effects analysis. McMullen (a) calculations use number of secondary cases and contacts identified from single transmission chains while (b) uses total secondary cases and contacts reported by each case and contact. Method (b) follows Fine et al methods. *CDC[43] and McMullen[39] do not report SARs that are stratified by vaccination status therefore overall household SARs was decidedly included in the forest plot*.

Four papers discussed SAR in monkeypox outbreaks, and their results are summarised in Fig 4. Three were analyses of active surveillance data from the time frames 1980-85, 1981-86 and 2005-7[14, 20, 39] and one was a MMWR situation report describing the 1996-7 Kasai outbreak in DRC.[43] One other paper provided sufficient information to calculate the SAR: a case report of a single case occurring in North Kivu, DRC, in 2012.[53]

Three papers reported ‘attack rates’ that did not meet the formal definition for either a primary or SAR.[57, 66, 70, 71] Due to the uncertainty of how these unspecified attack rate values were obtained or what they refer to, primary household attack rates, i.e. the proportion of households with a suspected or confirmed case of monkeypox within the occupancy presenting within the incubation period of confirmed or suspected cases, could not be analysed. An ‘attack rate in a single household’ of 71% was reported in the 2017-18 Nigeria outbreak although it is unclear what values informed the numerator and denominator. This reflects a single household only and cannot be taken as more than a chance finding.[57]

Overall, SAR ranges suggest between 0-11% of unvaccinated contacts of primary cases may become clinical cases during an outbreak. The pooled estimate for unvaccinated household contacts is in the order of 8%.

Four papers discuss the number of generations of transmission observed in monkeypox outbreaks. Reports of up to 7 serial transmission events in the DRC and ROC have been consistently reported.[39, 66, 72] Two of these papers discussed nosocomial outbreak expansion.[72, 73]

The R_0_, or the number of secondary cases expected to arise from a single primary case in a naïve population was estimated in just two studies.[14, 39] Analysis of active surveillance data collected in the DRC between 1980 and 1984 calculated a basic reproduction number of 0.8.[14] (Cohort n = 209 cases and contacts, assuming an average of 10.7 susceptible contacts per primary case). The net reproduction number R_net_ was estimated to be 0.3 cases in this population. When the upper confidence interval limit for the crude SAR was taken, the R_0_ was 1.0 indicating the possibility of persistence in human populations could not be excluded. Using the same methods as Fine et al, McMullen calculated an R_net_ of 0.6 for 2005-2007 study data collected in Sankuru District in DRC. (N = 703 cases and contacts, average 6.2 secondary contacts).[39]

#### Case fatality

Thirty documents reported outbreak or annual CFR across 11 countries (see Table 1). Insufficient demographic data meant stratification by vaccination status, age, or gender was not possible. As a result, the CFR represent heterogeneous populations and data collection methods (active case finding, ISDR notifiable reporting) therefore it would not be appropriate to pool or combine values.

**Table 1:** CFR by country and year.

Country	Location	Time Frame	Total suspected cases	Total deaths	CFR
DRC	Basankusu Territory[38]	1970	1	1*	100%
	Various – national total[67]	1981-86	338	33^§^	9.8%
	Katako-Kombe HZ[43]	02/1996-02/1997	92	3	3.3%^†^
	Kasai Oriental[43]	02/1996 – 10/1997	511	5	1.5% ^¶^
	Katako-Kombe, Lodja Nord, Sud HZ[43]	03/1997–05/1997	170	0	0%
	Sankuru[14]	1999	ND	315	ND
	Equateur Province[16]	02/2001-08/2001	23	5	21.7%
	Businga[75]	2002	72	7	9.7%
	Bokungu HZ[66]	2013	99	10	10.1%
	Ateki HZ[41]	1/1/2016–1/3/2016	155	11	7.1%
	Sankuru[42]	01/01/2018–08/07/2018	2995	36	1.20%
DRC	NA (ISDR data)[64]	2001	388	13	3.4%
		2002	881	14	1.6%
		2003	755	16	2.1%
		2004	1024	29	2.8%
		2005	1708	26	1.5%
		2006	783	20	2.6%
		2007	970	11	1.1%
		2008	1599	67	4.2%
		2009	1919	27	1.4%
		2010	2322	26	1.1%
		2011	2208	15	0.7%
		2012	2629	34	1.3%
		2013	2460	37	1.5%
CAR	Pimu CAR/DRC border[49, 50]	14/08/2001	8	2	25%
	Deep forest, Southern CAR[49]	06/2010	2	0	0%^¶^
	Bria[50]	30/01/2015:	3	1	33%
	Mbomou province[46]	04/12/2015–02/2016	10	2	20%
	Haute-Kotto health district[46]	04/09/16-07/10/2016	26	1	3.8%****
	Alindao-Mingala Health District[54]	08/2016-10/2016	26	2	7.7%
	M’baïki district[48]	12/04/2017	2	0	0%
	Bangassou, sub-district Rafai[76]	29/03/2017	15	1	6.7%
	Bambari, Ippy sub-district[42]	17/03/2018	9	0	0%
	Mbaïki: Bangandou sub-district[42]	30/06/2018	5	0	0%
ROC	Likouala department^[^^52^^,^ ^73^^,^ ^77^^]^	15/04/03-23/06/2003	12	1	8.3%^§§^
		04/10-11/2010	11	1	9.1%
		18/01/17 – 15/10/17	88	6	6.8%
Sudan	Unity State[56]	10/2005	37	0	0%
Cameroon	ND[45]	1989	1	0	0
	Njikwa Health District[78]	30/04/2018	6	0	0%
Gabon	Lambarene[74]	1987	1	1	100%^††^
	Region between Lamberene and N’Djole[44]	01/1991-06/ 1991	9	0	0%
Nigeria	Abia State[79]	1971	2	0	0%
	Oyo State[79]	1978	1	0	0%
	24 States[57]	2017-18	228	6**	2.6%
Sierra Leone	Moyamba District[79]	1970-71	1	0	0%
	Pujehun district[77]	14/03/2017	1	0	0%
Liberia	ND[79]	1970-71	4	0	0%
Cote d’Ivoire	ND[37]	10/1971	1	0	0%
USA	Multiple States – import from Ghana[62, 80]	2003	47	0^¶^	0%

ND = Not Described. Green = WA strain. Orange = CB Strain.

*Died of Measles.

^§^All deaths in unvaccinated children aged 3 months-8YO. CFR <2YO = 20%, <4YO: 15%, 5-9YO: 6.6%. CFR in unvaccinated primary cases = 11.8% vs unvaccinated secondary case = 9.6%.

^†^3 (3%) of 92 patients died; all <3YO. The other three deaths reported during the initial investigation were not monkeypox cases or occurred in a village in which no active case search was conducted during follow-up.

^¶^Out of 344 cases identified in Katako-Kombe HZ.

**N = 4/6 were immunocompromised.

^§§^N = 1 unclear cause; post-operative peritoneal infection after exploratory surgery, during acute monkeypox. N = 1 infant did not regain full health and died 6 months later with reported haemolytic anaemia, details unclear. N = 1 experienced viral conjunctivitis 6 weeks post-monkeypox with extensive corneal damage.

^††^9-month old girl co-infected with Plasmodium falciparum.

^¶¶^Non-fatal encephalitis occurred in a 6YO girl.

The often-quoted 10% CFR number comes from early 1981-6 data: of 33 deaths out of 338 cases during this time (CFR 9.8%); all deaths occurred in unvaccinated individuals. In contrast, ISDR data from the DRC between 2001 and 2013 show overall CFR are consistently <5%. CFR from countries with the WA virus were mostly 0% for outbreaks with 1-4 cases. The CFR for the Nigeria outbreak was 2.8% (6 deaths, 4 of whom were immunocompromised, out of 228 suspect cases). The CFR was 0% in the US outbreak (47 cases). No deaths have been reported from the 04/2018 outbreak in Cameroon, or from the most recent outbreak in CAR.

Where causes of death were documented, infants, young children <10, pregnant women, patients with complications and immunocompromised individuals were particularly implicated, suggesting these are high-risk groups.[38, 57, 67, 73, 74]

### Case definitions

‘Suspected case’ definitions were described in 20 documents (see S4 Table for definitions by situations and purpose). Two papers were identified in which case definitions were evaluated; one was a conference abstract.[31, 81] Surveillance case definitions were largely non-specific and highly sensitive to ensure all possible monkeypox cases are detected to trigger an outbreak investigation and estimate total annual monkeypox case burden. These are subject to a high number of false positive cases. Outbreak case definitions were more specific to monkeypox symptoms and aimed to distinguish between monkeypox and other rash-like illnesses namely Varicella Zoster Virus (VZV).

McCollum[31] and others evaluated one surveillance suspected case definition used by the DRC Ministry of health (MOH) in endemic areas:

“*an individual with* ‘*febrile prodrome and either the presence of a) vesicular or pustular rash or scars on face, palms, and soles*, *or b) the presence of 5 or more variola-like scar*” [31]

Sensitivity was 100% and specificity was 80% when applied to clinical symptoms for 6 suspected cases for which laboratory data were also available. The 6 cases came from a total sample of 33 suspect cases from multiple unspecified African countries submitted to CDC, Atlanta.

In a larger study Osadebe[81] et al evaluated the aforementioned plus a second MOH definition used at outbreak level:

“*Individuals who have a vesicular or pustular eruption with deep-seated, firm pustules and at least one of the following a) febrile prodrome*, *b) lymphadenopathy (inguinal, axillary, or cervical), and/or c) pustules of crusts on the palms of the hands or soles of the feet*”.

Both definitions were applied to a 2009-2014 cohort of 645 individuals that had met the surveillance criteria for monkeypox, and had laboratory results available. Both the ‘detection’ and ‘discriminatory’ definition had high sensitivities of 93% and 98% but low specificities of 9% and 26% respectively. Clinical symptoms such as lymphadenopathy, fatigue and rash characteristics such as deep-seated, same-size lesions, present on the arms, legs, palms and soles showed ≥80% sensitivity for monkeypox. Nausea, conjunctivitis, lesions on genitals or bedridden were symptoms with ≥80% specificity but only occurred in 32% of monkeypox patients. Notably, lesions on palms and soles occurred in 91.2% vs. 81.3% and lymphadenopathy occurred in 85.3% vs. 71.4% of laboratory-confirmed monkeypox and laboratory-confirmed VZV cases, respectively.

A ‘Receiver operator characteristic’ (ROC) analysis calculated sensitivity and specificity for cases with febrile prodrome plus up to 12 signs or symptoms (see S4 Table). Those with febrile prodrome plus <7 signs/symptoms had high sensitivities (>90%) but low specificities (<40%). Those with febrile prodrome plus >8 signs/symptoms had higher specificities (>90%) but low sensitivities (<40%). Seven to 8 signs/symptoms showed an optimum combination of sensitivity and specificity. Maximum specificity achieved was 71%.

Operational VZV case definitions were not provided for comparison in almost all outbreak reports or case definition evaluation studies. Laudisoit[41] et al designed and tested a suspect human monkeypox score (range 1-6) corresponding to the presence of up to 7 symptoms with variable weightings. Results showed little difference between monkeypox and VZV scores (VZV patients score: 2.5-4, Vs monkeypox score: 4-4.5).

Another study highlighted cases of MPX/VZV co-infection in a suspected monkeypox cohort identified through active surveillance between 2006-8 in Sankuru, DRC. Fewer symptoms were noted in co-infected cases compared with monkeypox-only cases further complicating clinical distinction.[82]

### Management

This review found a lack of detail on case management, usually of secondary skin infections, in the published literature. Just 8 documents mentioned antibiotic case management anecdotally.[41, 49, 50, 53, 62, 80] NCDC have not yet published about the clinical presentations and case management from the recent outbreak.

## Discussion

The literature that is available provides an incomplete picture of monkeypox outbreak patterns over time. Data were mostly insufficient for statistical analysis and as a result the conclusions that could be drawn were general, as are recommendations. Most available data came from the DRC as the only country in Central and West Africa to include monkeypox as a notifiable disease. ISDR data itself are likely to underestimate the true case burden due to exclusion of patients who use private clinics and traditional healers or do not access clinical sentinel surveillance sites. Outside the DRC, focusing on single outbreaks to analyse changes in epidemiology is likely to introduce a degree of ‘outbreak bias’ whereby the outlier monkeypox outbreaks receive extra attention but are less representative of usual conditions.[83]

Evidence supporting an increase in monkeypox incidence over time was most consistent in the DRC where incidence data for other diseases remain unchanged whilst monkeypox incidence increased over time, i.e. suggesting this is not only as a result of rising population size.[1, 2, 39, 64, 84] The increased number of outbreaks reported outside the DRC suggest a rise in incidence has occurred over a wider geography. There have been more outbreaks reported in the last 5 years in the CAR than in all years prior. Although there have been some improvements in surveillance systems following the Global Health Security Agenda and joint external evaluation assessments as well as some improvements in laboratory capacity, it is unlikely to be the sole cause underpinning the changes seen in monkeypox epidemiology.[1]

It is plausible and logical that the increase in reported monkeypox cases is a consequence of increased population density, encroachment of human settlements into unknown animal reservoirs, or an increase in the population of susceptible individuals since the cessation of the smallpox vaccination programme. Almost all individuals under the age of 40 are unvaccinated in the DRC.[2, 15]Additionally, immunity of previously vaccinated individuals has waned. Data required to support these explanations including longitudinal population density data at the province level, maps of population expansion, conclusive animal reservoir studies, and accurate annual outbreak data at the same province level were not available in the accessible literature. As a result, it is difficult to substantiate these explanations with strong evidence.

The apparent increase in the average age of monkeypox cases in the DRC over time is likely to reflect the increase in the average age of susceptible individuals born after the discontinuation of smallpox vaccination, in 1980.[2, 15] Concentration of cases in children has been speculated to reflect their playing with animals or activities of young male hunters coming into frequent contact with sylvatic animals.[40] Further studies would help characterise behavioural associations in demographic groups.

From the few virus sample studies available, there have been no findings to suggest new virus strains are responsible for re-emerging outbreaks in Cameroon, Nigeria or the CAR. Overall, samples are seldom taken, and very few cases are laboratory-confirmed. Improvements in field team and laboratory capacity to routinely collect and test lesion samples would greatly help the literature; consistent case confirmation would provide more accurate estimates of true monkeypox outbreak occurrence and their respective case burden and further studies of virulence loci would improve knowledge of the virus.

The pattern of cases in the 2017-18 Nigeria outbreak suggests multiple local zoonotic introductions with a strain familiar to Nigeria. Further investigation would be necessary to address the possibility of a widespread zoonotic reservoir. Similarly, data collected on clinical features stratified by region, population demographic and strain would improve understanding of clinical differences between the two clades as they present in local populations as well as informing local clinical case definition.

Without a known reservoir, a local ‘implicated-species list’ may help to inform risk reduction advice given in outbreaks, with regards to behaviours that pose risk of animal-to-human transmission. Hunting, handling, preparing and consuming bushmeat was consistently implicated in developing clinical monkeypox, a finding that did not appear to have changed over time with the exception of a single case-control study.[8, 40, 67] This study analysed just 15 primary cases – larger case-control studies with greater power are necessary.[40] We recommend focus on strengthening outbreak teams to investigate index case interaction with wildlife to build local knowledge.

The SAR data on which secondary transmission knowledge is based were surprisingly sparse. SARs in unvaccinated household contacts were in the order of 10%. The differences in ‘crude’ SARs between 1981-86, 1996-7 and 2005-7 data were noticeable and likely reflect an increase in unvaccinated individuals in the population. SARs are likely to be overestimates due to case ascertainment bias in outbreak investigations. Contact tracing was carried out one year after the outbreak in 2 studies.[39, 43] These SARs carry a high risk of recall bias. Both R_0_’s and SARs are a function of context including degree of close contact in the community, hygiene levels, knowledge of the condition and whether intervention occurred. These values may not be applicable to the WA strain or either strain outside their endemic context, for example in a crowded urban environment. Routine contact tracing in outbreak investigations would provide denominator values with which up-to-date SARs could be calculated. Data collection to provide household SAR values from a wider range of endemic areas, across both clades of virus is recommended to better inform outbreak response strategies. Generations of up to 7 transmission events indicate that monkeypox should not be underestimated during an outbreak in the CB setting.[39, 66, 72, 73] The frequency of nosocomial transmission to both healthcare workers and well individuals in the vicinity highlight the necessity for PPE.[50, 54, 72, 73]

The CFR for the CB strain was consistently higher than the WA strain which appeared to be non-fatal until the 2017-18 Nigeria outbreak.[57] Fatalities specifically in infants, young children <10, pregnant women, patients with complications and immunocompromised individuals were highlighted.[38, 57, 67, 73, 74] CFR data were otherwise limited by lack of detail to allow stratification by vaccination status, sex, age or immune status. We cannot conclude from the limited data available whether there has been a change in CFR since the cessation of smallpox vaccination. More data is needed to identify cause of death where fatalities do occur, and in which patient demographic.

A variety of suspected case definitions used in monkeypox surveillance and outbreak investigation were highlighted. Just two definitions have been formally evaluated for sensitivity and specificity. Specificity is contextual and reflects presence of other rash-like illnesses in the population while sensitivity can be influenced by the clinical presentation of monkeypox in a given population. This in turn is influenced by virus strain and baseline population health; level of malnutrition, co-infections and HIV prevalence. This means case definitions are unlikely to apply with the same effectiveness to different geographical contexts and strains. As a result, case definitions might need to be developed and evaluated more locally rather than using a common monkeypox case definition across multiple regions. With regards to purpose, sensitive definitions could identify almost all monkeypox cases to isolate and minimise transmission, but low specificity risks over-diagnosis and over-burdening healthcare facilities in low-resource settings. Summative symptom criteria may be optimal in reducing over-diagnosis but is likely to compromise sensitivity and may prove too complicated a definition to implement. Further surveillance and outbreak case definition evaluation studies would be beneficial in informing local operational use. Consistent denotation of which case definition was used in an outbreak is also recommended.

Given the clinical overlap between monkeypox and VZV, a proportion of the identified but unconfirmed monkeypox cases reported in the literature are likely to be VZV. As a result, the conclusions drawn about monkeypox trends from surveillance figures may be subject to inaccuracies. Accessible and affordable point of care rapid diagnostic testing would allow reliable discrimination between the two diseases for surveillance and outbreak reporting purposes.

In low-resource endemic settings, when deciding whether to hospitalise or isolate in the community, hospital-associated risks[54, 72, 73] must be balanced with the risk of household transmission. Unvaccinated household members, nature of likely person-to-person contact, level of hygiene, and ability to isolate in a different room should all be considered. Prioritising suspected cases in the aforementioned ‘high-risk groups’ for hospitalisation may be appropriate, alongside applying isolation and behavioural risk-reduction advice in the community for lower-risk individuals. Monkeypox and HIV co-infection was another area notably lacking research. Use of smallpox vaccine could be a possibility when HIV population prevalence, or testing is available, and knowledge of co-infection is improved.

Outbreak studies would benefit from including descriptions of the response imposed. This would allow analysis of outbreak morbidity measures by intervention and could guide future recommendations to local teams. Similarly, there is a dearth of data regarding clinical interventions, which may be useful in more remote endemic contexts. Evidence informing routine use of antibiotics in secondary cutaneous infection prophylaxis was anecdotal and insufficient to form conclusions about the effectiveness or normative use of antibiotics in case management. Furthermore, recognition and support of current local research efforts into monkeypox will allow larger research undertaking and presentation of results into the wider literature.

While monkeypox virus has not established and propagated itself in the human population since the cessation of smallpox vaccination, the risks to populations in endemic areas are evident. Significant improvements in the quality and quantity of outbreak data collection are urgently needed to improve the monkeypox research portfolio to inform appropriate case management and public health response.

## Supporting information

S1 Text Search Strategy: EMBASE (OVID). (DOCX)

S1 Table Inclusion/Exclusion criteria. (DOCX)

S2 Table Age and sex characteristics by country and year. (DOCX)

S3 Table Summary of study findings on risk factors for primary introduction of monkeypox. (DOCX)

S4 Table Case definition summary. (DOCX)

S5 Table Secondary attack rate table. (DOCX)

S6 Table Suspected, confirmed and fatal monkeypox cases by country and year. (DOCX)

## References

[1] Durski KN, McCollum AM, Nakazawa Y, Petersen BW, Reynolds MG, Briand S, et al Emergence of Monkeypox - West and Central Africa, 1970-2017. Mmwr. 2018;Morbidity and mortality weekly report. 67(10):306–10. 10.15585/mmwr.mm6710a5 . 29543790 PMC5857192

[2] Rimoin AW, Mulembakani PM, Johnston SC, Lloyd Smith JO, Kisalu NK, Kinkela TL, et al Major increase in human monkeypox incidence 30 years after smallpox vaccination campaigns cease in the Democratic Republic of Congo. Proceedings of the National Academy of Sciences of the United States of America. 2010;107(37):16262–7. 10.1073/pnas.1005769107 . 20805472 PMC2941342

[3] Marennikova SS, Seluhina EM, Malceva NM, Ladnyj ID. Poxviruses isolated from clinically ill and asymptomalically infected monkeys and a chimpanzee. Bulletin of the World Health Organization. 1972;46(5):613–20. . 4340220 PMC2480788

[4] Pv Magnus, Andersen EK Petersen KB, Birch-Andersen A. A Pox-like Disease in Cynomolgus Monkeys. Acta Pathologica Microbiologica Scandinavica. 1959;46(2):156–76. 10.1111/j.1699-0463.1959.tb00328.x

[5] Doty JB, Malekani JM, Kalemba LN, Stanley WT, Monroe BP, Nakazawa YU, et al Assessing Monkeypox Virus Prevalence in Small Mammals at the Human-Animal Interface in the Democratic Republic of the Congo. Viruses. 2017;9(10):03 10.3390/v9100283 . 28972544 PMC5691634

[6] Hutin YJ, Williams RJ, Malfait P, Pebody R, Loparev VN, Ropp SL, et al Outbreak of human monkeypox, Democratic Republic of Congo, 1996 to 1997. Emerging Infectious Diseases. 2001;7(3):434–8. 10.3201/eid0703.010311 . 11384521 PMC2631782

[7] Khodakevich L, Jezek Z, Messinger D. Monkeypox virus: ecology and public health significance. Bulletin of the World Health Organization. 1988;66(6):747–52. . 2853010 PMC2491157

[8] Jezek Z, Grab B, Szczeniowski M, Paluku KM, Mutombo M. Clinico-epidemiological features of monkeypox patients with an animal or human source of infection. Bulletin of the World Health Organization. 1988;66(4):459–64. 2844428 PMC2491168

[9] Reynolds MG, Carroll DS, Olson VA, Hughes C, Galley J, Likos A, et al A silent enzootic of an orthopoxvirus in Ghana, West Africa: Evidence for multi-species involvement in the absence of widespread human disease. American Journal of Tropical Medicine and Hygiene. 2010;82(4):746–54. 10.4269/ajtmh.2010.09-0716 . 20348530 PMC2844556

[10] Jezek Z, Szczeniowski M, Paluku KM, Mutombo M. Human monkeypox: clinical features of 282 patients. Journal of Infectious Diseases. 1987;156(2):293–8. 10.1093/infdis/156.2.293 . 3036967

[11] Mbala PK, Huggins JW, Riu-Rovira T, Ahuka SM, Mulembakani P, Rimoin AW, et al Maternal and Fetal Outcomes among Pregnant Women with Human Monkeypox Infection in the Democratic Republic of Congo. Journal of Infectious Diseases. 2017;216(7):824–8. 10.1093/infdis/jix260 . 29029147

[12] Withers MR, Kingebeni PM, Muyembe JJT, Martin J, Riu-Rovira T, Huggins J, et al Outcome of four pregnancies in congolese women with monkeypox infection. American Journal of Tropical Medicine and Hygiene. 2011;1):397 .

[13] Kisalu NK, Mokili JL. Toward Understanding the Outcomes of Monkeypox Infection in Human Pregnancy. Journal of Infectious Diseases. 2017;216(7):795–7. 10.1093/infdis/jix342 . 29029238 PMC6279131

[14] Fine PE, Jezek Z, Grab B, Dixon H. The transmission potential of monkeypox virus in human populations. International Journal of Epidemiology. 1988;17(3):643–50. 10.1093/ije/17.3.643 . 2850277

[15] Heymann DL, Szczeniowski M, Esteves K. Re-emergence of monkeypox in Africa: a review of the past six years. British Medical Bulletin. 1998;54(3):693–702. 10.1093/oxfordjournals.bmb.a011720 . 10326294

[16] Meyer H, Perrichot M, Stemmler M, Emmerich P, Schmitz H, Varaine F, et al Outbreaks of disease suspected of being due to human monkeypox virus infection in the Democratic Republic of Congo in 2001. Journal of Clinical Microbiology. 2002;40(8):2919–21. 10.1128/JCM.40.8.2919-2921.2002 . 12149352 PMC120683

[17] Mutombo M, Arita I, Jezek Z. Human monkeypox transmitted by a chimpanzee in a tropical rain-forest area of Zaire. Lancet. 1983;1(8327):735–7. Epub 1983/04/02. 10.1016/s0140-6736(83)92027-5 . 6132084 PMC9534202

[18] Reynolds MG, Yorita KL, Kuehnert MJ, Davidson WB, Huhn GD, Holman RC, et al Clinical manifestations of human monkeypox influenced by route of infection. Journal of Infectious Diseases. 2006;194(6):773–80. 10.1086/505880 . 16941343

[19] Jezek Z, Grab B, Paluku KM, Szczeniowski MV. Human monkeypox: Disease pattern, incidence and attack rates in a rural area of northern Zaire. Tropical and Geographical Medicine. 1988;40(2):73–83. . 2841783

[20] Jezek Z, Grab B, Szczeniowski MV, Paluku KM, Mutombo M. Human monkeypox: secondary attack rates. Bulletin of the World Health Organization. 1988;66(4):465–70. . 2844429 PMC2491159

[21] Parker S, Nuara A, Buller RM, Schultz DA. Human monkeypox: an emerging zoonotic disease. Future Microbiology. 2007;2(1):17–34. 10.2217/17460913.2.1.17 . 17661673

[22] Likos AM, Sammons SA, Olson VA, Frace AM, Li Y, Olsen-Rasmussen M, et al A tale of two clades: Monkeypox viruses. Journal of General Virology. 2005;86(10):2661–72. . 16186219 10.1099/vir.0.81215-0

[23] Panning M, Asper M, Kramme S, Schmitz H, Drosten C. Rapid detection and differentiation of human pathogenic orthopox viruses by a fluorescence resonance energy transfer real-time PCR assay. Clinical Chemistry. 2004;50(4):702–8. 10.1373/clinchem.2003.026781 . 14962998

[24] Neubauer H, Reischl U, Ropp S, Esposito JJ, Wolf H, Meyer H. Specific detection of monkeypox virus by polymerase chain reaction. Journal of Virological Methods. 1998;74(2):201–7. 10.1016/s0166-0934(98)00099-8 . 9779620

[25] Li Y, Olson VA, Laue T, Laker MT, Damon IK. Detection of monkeypox virus with real-time PCR assays. Journal of Clinical Virology. 2006;36(3):194–203. 10.1016/j.jcv.2006.03.012 . 16731033 PMC9628957

[26] Jezek Z, Szczeniowski M, Paluku KM, Mutombo M, Grab B. Human monkeypox: Confusion with chickenpox. Acta Tropica. 1988;45(4):297–307. . 2907258

[27] Hoff NA, Morier DS, Kisalu NK, Johnston SC, Doshi RH, Hensley LE, et al Varicella Coinfection in Patients with Active Monkeypox in the Democratic Republic of the Congo. EcoHealth. 2017;14(3):564–74. 10.1007/s10393-017-1266-5 . 28894977

[28] MacNeil A, Reynolds MG, Carroll DS, Karem K, Braden Z, Lash R, et al Monkeypox or varicella? lessons from a rash outbreak investigation in the republic of the congo. American Journal of Tropical Medicine and Hygiene. 2009;80(4):503–7. . 19346366

[29] Reynolds MG, McCollum AM, Nguete B, Lushima RS, Petersen BW. Improving the care and treatment of monkeypox patients in low-resource settings: Applying evidence from contemporary biomedical and smallpox biodefense research. Viruses. 2017;9 (12) (no pagination)(380). . 10.3390/v9120380 PMC5744154 29231870

[30] Moher D, Liberati A, Tetzlaff J, Altman DG, The PG. Preferred Reporting Items for Systematic Reviews and Meta-Analyses: The PRISMA Statement. PLOS Medicine. 2009;6(7):e1000097 10.1371/journal.pmed.1000097 19621072 PMC2707599

[31] McCollum AM, Balilo MP, Pukuta E, Muyembe JJ, Damon IK, Reynolds MG. Towards enhanced surveillance for monkeypox: Application of a robust clinical case definition. American Journal of Tropical Medicine and Hygiene. 2010;1):122 10.4269/ajtmh.2010.09-0738 . 20595490 PMC2912588

[32] Guagliardo SA, Reynolds M, Shongo RL, Wemakoy O, McCollum A. A comparison of three statistical thresholds to trigger a public health response to monkeypox-Democratic Republic of the Congo, 2011-13. American Journal of Tropical Medicine and Hygiene. 2017;97 (5 Supplement 1):281 .28719336

[33] Laudisoit A, Verheyen E, Baelo P, Akonda I, Nebesse C, Ngoy S, et al A One Health team to improve Monkeypox virus outbreak response: An example from the Democratic Republic of the Congo. Tropical Medicine and International Health. 2017;22 (Supplement 1):53 .

[34] Laudisoit A, Baelo P, Mussaw Awazi M, VanHoutte N, VanHees M, Amundala N, et al Biodiversity, Bushmeat and Monkeypox in the Democratic Republic of the Congo: Another viral threat upon larger cities? Tropical Medicine and International Health. 2015;1):30–1. .

[35] Population by age and sex (thousands) (DRC). [Internet]. Population Division. 2017.

[36] Jezek Z, Marennikova SS, Mutumbo M, Nakano JH, Paluku KM, Szczeniowski M. Human monkeypox: a study of 2,510 contacts of 214 patients. Journal of Infectious Diseases. 1986;154(4):551–5. 10.1093/infdis/154.4.551 . 3018091

[37] Breman JG, Nakano JH, Coffi E, Godfrey H, Gautun JC. Human poxvirus disease after smallpox eradication. American Journal of Tropical Medicine & Hygiene. 1977;26(2):273–81. 10.4269/ajtmh.1977.26.273 . 192091

[38] Ladnyj ID, Ziegler P, Kima E. A human infection caused by monkeypox virus in Basankusu Territory, Democratic Republic of the Congo. Bulletin of the World Health Organization. 1972;46(5):593–7. . 4340218 PMC2480792

[39] McMullen CL, Mulembekani P, Hoff NA, Doshi RH, Mukadi P, Shongo R, et al Human monkeypox transmission dynamics thirty years after smallpox eradication in the Sankuru district, democratic republic of Congo. American Journal of Tropical Medicine and Hygiene. 2015;93 (4 Supplement):341 .

[40] Nolen LD, Osadebe L, Katomba J, Likofata J, Mukadi D, Monroe B, et al Introduction of Monkeypox into a Community and Household: Risk Factors and Zoonotic Reservoirs in the Democratic Republic of the Congo. American Journal of Tropical Medicine & Hygiene. 2015;93(2):410–5. 10.4269/ajtmh.15-0168 . 26013374 PMC4530773

[41] Laudisoit A. Bushmeat and Monkeypox: Yahuma Health Zone – Aketi Health Zone - Bombongolo Health Area. Kisangani, DRC: CIFOR, Université de Kisangani, DRC, 2016.

[42] WHO. WHO AFRO Outbreaks and Other Emergencies, Week 30: 21 -27 July 2018 (Data as reported by 17:00; 27 July 2018). Reliefweb: 2018.

[43] Prevention. CfDCa. Human monkeypox -- Kasai Oriental, Democratic Republic of Congo, February 1996-October 1997. MMWR - Morbidity & Mortality Weekly Report. 1997;46(49):1168–71. . 9408046

[44] Record. WE. Monkeypox, 1991. Gabon. Weekly Epidemiological Record. 1992;67(14):101–2. . 1314067

[45] Tchokoteu PF, Kago I, Tetanye E, Ndoumbe P, Pignon D, Mbede J. [Variola or a severe case of varicella? A case of human variola due to monkeypox virus in a child from the Cameroon]. Ann Soc Belg Med Trop. 1991;71(2):123–8. Epub 1991/06/01. . 1656900

[46] WHO. Monkeypox in Central African Republic. Reliefweb: 2016.

[47] Central African Republic: Monkey Pox Outbreak - Dec 2015 [Internet]. Reliefweb: IFRC; 2016

[48] WHO. WHO AFRO Outbreaks and Other Emergencies, Week 16: 15 – 21 April 2017 Data as reported by 17:00 21 April 2017. Reliefweb: 2017.

[49] Berthet N, Nakoune E, Whist E, Selekon B, Burguire AM, Manuguerra JC, et al Maculopapular lesions in the Central African Republic. The Lancet. 2011;378(9799):1354 . 10.1016/S0140-6736(11)61142-2 21982097

[50] Nakoune E, Selekon B, Komoyo GF, Kazanji M, Garba-Ouangole SM, Janssens C, et al A Nosocomial Outbreak of Human Monkeypox in the Central African Republic. Open Forum Infectious Diseases. 2017;4(4). 10.1093/ofid/ofx168 29732376 PMC5920348

[51] IFRC. Central African Republic: Monkey Pox Outbreak - Dec 2015. Reliefweb: 2016.

[52] Reynolds MG, Emerson GL, Pukuta E, Karhemere S, Muyembe JJ, Bikindou A, et al Short report: Detection of human monkeypox in the Republic of the Congo following intensive community education. American Journal of Tropical Medicine and Hygiene. 2013;88(5):982–5. 10.4269/ajtmh.12-0758 . 23400570 PMC3752768

[53] McCollum AM, Nakazawa Y, Ndongala GM, Pukuta E, Karhemere S, Lushima RS, et al Human monkeypox in a conflict region of the democratic republic of the Congo. American Journal of Tropical Medicine and Hygiene. 2013;1):17–8. . 10.4269/ajtmh.15-0095 PMC4596588 26283752

[54] Kalthan E, Tenguere J, Ndjapou SG, Koyazengbe TA, Mbomba J, Marada RM, et al Investigation of an outbreak of monkeypox in an area occupied by armed groups, Central African Republic. Medecine et Maladies Infectieuses. 2018;48(4):263–8. 10.1016/j.medmal.2018.02.010 . 29573840 PMC9533891

[55] Damon IK, Roth CE, Chowdhary V. Discovery of monkeypox in Sudan. New England Journal of Medicine. 2006;355(9):962–3. 10.1056/NEJMc060792 . 16943415

[56] Formenty P, Muntasir MO, Damon I, Chowdhary V, Opoka ML, Monimart C, et al Human monkeypox outbreak caused by novel virus belonging to Congo Basin clade, Sudan, 2005. Emerging Infectious Diseases. 2010;16(10):1539–45. 10.3201/eid1610.100713 . 20875278 PMC3294404

[57] NCDC. Situation Report: Monkeypox Outbreak in Nigeria. Abuja: 2018.

[58] Faye O, Pratt CB, Faye M, Fall G, Chitty JA, Diagne MM, et al Genomic characterisation of human monkeypox virus in Nigeria. The Lancet Infectious Diseases. 2018 . 10.1016/S1473-3099(18)30043-4 PMC9628790 29361427

[59] Kugelman JR, Johnston SC, Mulembakani PM, Kisalu N, Lee MS, Koroleva G, et al Genomic variability of monkeypox virus among humans, Democratic Republic of the Congo. Emerging Infectious Diseases. 2014;20(2):232–9. 10.3201/eid2002.130118 . 24457084 PMC3901482

[60] Nakazawa Y, Mauldin MR, Emerson GL, Reynolds MG, Lash RR, Gao J, et al A phylogeographic investigation of African monkeypox. Viruses. 2015;7(4):2168–84. 10.3390/v7042168 . 25912718 PMC4411695

[61] Nakazawa Y, Emerson GL, Carroll DS, Zhao H, Li Y, Reynolds MG, et al Phylogenetic and ecologic perspectives of a monkeypox outbreak, southern Sudan, 2005. Emerging Infectious Diseases. 2013;19(2):237–45. 10.3201/eid1902.121220 . 23347770 PMC3559062

[62] Reed KD, Melski JW, Graham MB, Regnery RL, Sotir MJ, Wegner MV, et al The Detection of Monkeypox in Humans in the Western Hemisphere. New England Journal of Medicine. 2004;350(4):342–50. 10.1056/NEJMoa032299 . 14736926

[63] Mukinda VB, Mwema G, Kilundu M, Heymann DL, Khan AS, Esposito JJ. Re-emergence of human monkeypox in Zaire in 1996. Monkeypox Epidemiologic Working Group. Lancet. 1997;349(9063):1449–50. 10.1016/s0140-6736(05)63725-7 . 9164323 PMC9533927

[64] Hoff N, Ilunga BK, Shongo R, Muyembe JJ, Mossoko M, Okitolonda E, et al Human monkeypox disease surveillance and time trends in The Democratic Republic of Congo, 2001-2013. American Journal of Tropical Medicine and Hygiene. 2014;1):339 .24343885

[65] Hoff NA, Kebela-Ilunga B, Eckhoff P, Mukadi P, Mossoko M, Muyembe-Tamfum JJ, et al A descriptive and quantitative analysis of potential underestimation of human monkeypox cases in the passive surveillance system in the democratic republic of congo. American Journal of Tropical Medicine and Hygiene. 2015;93 (4 Supplement):242 .

[66] Nolen LD, Osadebe L, Katomba J, Likofata J, Mukadi D, Monroe B, et al Extended human-to-human transmission during a monkeypox outbreak in the Democratic Republic of the Congo. Emerging Infectious Diseases. 2016;22(6):1014–21. 10.3201/eid2206.150579 . 27191380 PMC4880088

[67] Jezek ZaF F. Human monkeypox. Monographs in Virology. Karger, editor. Basel1988.

[68] Reynolds MG, Davidson WB, Curns AT, Conover CS, Huhn G, Davis JP, et al Spectrum of infection and risk factors for human monkeypox, United States, 2003. Emerging Infectious Diseases. 2007;13(9):1332–9. 10.3201/eid1309.070175 . 18252104 PMC2857287

[69] Hoff N, Mulembakani PM, Johnston SC, Kisalu NK, Muyembe JJ, Hensley LE, et al Risk factors associated with human monkeypox in the democratic republic of Congo. American Journal of Tropical Medicine and Hygiene. 2014;1):199–200. .

[70] Kile JC, Fleischauer AT, Beard B, Kuehnert MJ, Kanwal RS, Pontones P, et al Transmission of monkeypox among persons exposed to infected prairie dogs in Indiana in 2003. Archives of Pediatrics and Adolescent Medicine. 2005;159(11):1022–5. 10.1001/archpedi.159.11.1022 . 16275790

[71] Croft DR, Sotir MJ, Williams CJ, Kazmierczak JJ, Wegner MV, Rausch D, et al Occupational risks during a monkeypox outbreak, Wisconsin, 2003. Emerging Infectious Diseases. 2007;13(8):1150–7. 10.3201/eid1308.061365 . 17953084 PMC2828073

[72] Jezek Z, Arita I, Mutombo M, Dunn C, Nakano JH, Szczeniowski M. Four generations of probable person-to-person transmission of human monkeypox. American Journal of Epidemiology. 1986;123(6):1004–12. 10.1093/oxfordjournals.aje.a114328 . 3010703

[73] Learned LA, Reynolds MG, Wassa DW, Li Y, Olson VA, Karem K, et al Extended interhuman transmission of monkeypox in a hospital community in the Republic of the Congo, 2003. American Journal of Tropical Medicine & Hygiene. 2005;73(2):428–34. . 16103616

[74] Muller G, Meyer A, Gras F, Emmerich P, Kolakowski T, Esposito JJ. Monkeypox virus in liver and spleen of child in Gabon. Lancet. 1988;1(8588):769–70. 10.1016/s0140-6736(88)91580-2 . 2895299

[75] DR Congo: MSF launches mass measles vaccination campaign in a country ravaged by catastrophic health situation. [Internet]. Reliefweb; 2002

[76] WHO AFRO Outbreaks and Other Emergencies, Week 13: 25 – 31 March 2017 Data as reported by 17:00 31 March [Internet]. Reliefweb: WHO; 2017

[77] WHO. WHO AFRO Outbreaks and Other Emergencies, Week 13: 25 – 31 March 2017 Data as reported by 17:00 31 March. Reliefweb: 2017.

[78] WHO. Monkeypox – Cameroon. 2018.

[79] Breman JG, Kalisa R, Steniowski MV, Zanotto E, Gromyko AI, Arita I. Human monkeypox, 1970-79. Bulletin of the World Health Organization. 1980;58(2):165–82. . 6249508 PMC2395797

[80] Sejvar JJ, Chowdary Y, Schomogyi M, Stevens J, Patel J, Karem K, et al Human monkeypox infection: A family cluster in the Midwestern United States. Journal of Infectious Diseases. 2004;190(10):1833–40. 10.1086/425039 . 15499541

[81] Osadebe L, Hughes CM, Shongo Lushima R, Kabamba J, Nguete B, Malekani J, et al Enhancing case definitions for surveillance of human monkeypox in the Democratic Republic of Congo. PLoS Neglected Tropical Diseases. 2017;11 (9) (no pagination)(e0005857). . 10.1371/journal.pntd.0005857 PMC5593177 28892474

[82] Hughes CM, Liu L, McCollum AM, Davidson W, Monroe B, Carroll D, et al Co-infections with monkeypox and varicella zoster viruses, the democratic republic of the Congo, 2010-2013. American Journal of Tropical Medicine and Hygiene. 2015;93 (4 Supplement):444 .

[83] Zell ER, Fine PEM. Outbreaks in Highly Vaccinated Populations: Implications for Studies of Vaccine Performance. American Journal of Epidemiology. 1994;139(1):77–90. 10.1093/oxfordjournals.aje.a116937 8296777

[84] Reynolds MG, Damon IK. Outbreaks of human monkeypox after cessation of smallpox vaccination. Trends in Microbiology. 2012;20(2):80–7. 10.1016/j.tim.2011.12.001 . 22239910

