# Supplementary material for: A systematic review of the epidemiology of human monkeypox outbreaks and implications for outbreak strategy
Source: PLoS Negl Trop Dis. 2019 Oct 16;13(10):e0007791. doi: 10.1371/journal.pntd.0007791 (PMC6816577; doi:10.1371/journal.pntd.0007791)
Supplement: S1 Text — (DOCX) [file pntd.0007791.s001.docx]

## S1 Text. Search Strategy.

1. Exp monkeypox/
2. Monkeypox virus/
3. 1 or 2
4. Limit 3 to English language
