# Supplementary material for: A systematic review of the epidemiology of human monkeypox outbreaks and implications for outbreak strategy
Source: PLoS Negl Trop Dis. 2019 Oct 16;13(10):e0007791. doi: 10.1371/journal.pntd.0007791 (PMC6816577; doi:10.1371/journal.pntd.0007791)
Supplement: S1 Table — (DOCX) [file pntd.0007791.s002.docx]

## S1 Table. Inclusion/Exclusion Criteria.

| Inclusion Criteria | Studies pertaining to epidemiological characteristics of human monkeypox e.g. transmission dynamics, genetic characterisation of strains, case numbers, CFR |
| --- | --- |
|  | Studies reporting surveillance data or case definitions |
|  | Outbreak investigation or situation reports |
|  | Studies on transmission risk factors |
|  | Studies that discuss case management of clinical monkeypox |
| Exclusion Criteria | Broad topic of zoonoses/focus on smallpox/lack focus on monkeypox |
|  | Studies reporting clinical complications |
|  | Serology studies |
|  | Laboratory or experimental studies |
|  | Animal Serology Studies |
|  | Studies on non-clinical diagnostics |
|  | Antiviral case management |
|  | Prevention methods |
|  | Author Reply |
|  | Literature Reviews |
