# Supplementary material for: A systematic review of the epidemiology of human monkeypox outbreaks and implications for outbreak strategy
Source: PLoS Negl Trop Dis. 2019 Oct 16;13(10):e0007791. doi: 10.1371/journal.pntd.0007791 (PMC6816577; doi:10.1371/journal.pntd.0007791)
Supplement: S2 Table — (DOCX) [file pntd.0007791.s003.docx]

## S2 Table. Age and Sex Characteristics by Country and Year

| **Country** | **Year(s)** | **Median age (mean) in years, or single age** | **Proportion male** | **Total number of suspected cases** |
| --- | --- | --- | --- | --- |
| **DRC** | 1970-79 [1] | 4‖ | 53% | 38 |
|  | 1981-86 [2, 3] | 4 (7) ‡‡ | 54%§ | 338 |
|  | 1996-97 [4] | 10¶ | 55% | 92 |
|  | 2001-4 [5] | 11 (15) | 49%* | 134 |
|  | 2005-7 [6] | 10 (12) | 62% | 760 |
|  | 2013 [7] | 10 (16) | 57%†† | 63 |
|  | 2011-14 [8] | 15.5 (14) | 67% | 6 |
|  | 2008-14 [9] | 13 (17) | 53% | 1025 |
| **CAR** | 2010 [10] | 14.5 | 100% | 2 |
|  | 2015 [11] | 7 | ND | 2 |
|  | 2016 [12] | 24 | 54% | 26 |
|  | 2017 [13] | 35 | 100% | 1 |
|  | 2017 [14] | 27.5 (24) | 50% | 10 |
| **ROC** | 2003 [15] | 10 | 67% | 12 |
|  | 2013 [16] | 8.5† | 60% | 10 |
|  | 2017 [13] | ND** | 49% | 70 |
| **Nigeria** | 1971 [1] | 4 | 0% | 1 |
|  | 2017-18 [17] | 30 | 69% | 228 |
| **Cote D’Ivoire** | 1971 [18] | 5 | 100% | 1 |
| **Sierra Leone** | 1970-71 [1] | 24 | 100% | 1 |
| **Cameroon** | 2018 [19] | 13 | 56% | 16 |
| **Liberia** | 1970-71 [1] | 5 (6) | 50% | 4 |
|  | 2017 [20] | 5 | 100% | 1 |
| **Southern Sudan** | 2005 [21] | ND§§ | 47% | 19 |
| **Gabon** | 1987 [22] | 9/12 | 0% | 1 |
|  | 1991 [23] | ND‖ ‖ | ND | 5 |
| **USA** | 2003 [24] | ND‡ | 53% | 47 |

ND = Not Described *Data on sex and age were available for 134/2734 suspect patients. †Data known for n=7 ‡n=8<18, n=29>18 §142/245 (58%) primary cases male, 40/93 (43%) secondary cases male ‖85%<15 ¶83%<15 **60%<15 ††Information available for 63/99 suspect cases. 11/17 known primary cases male (65%) ‡‡93%<15 §§Known ages: 5, 11, 13, 30, 32. 15/19 (79%) <20 years ‖ ‖Age range 3-11.

1. Breman JG, Kalisa R, Steniowski MV, Zanotto E, Gromyko AI, Arita I. Human monkeypox, 1970-79. Bulletin of the World Health Organization. 1980;58(2):165-82. PubMed PMID: 6249508.

2. Heymann DL, Szczeniowski M, Esteves K. Re-emergence of monkeypox in Africa: a review of the past six years. British Medical Bulletin. 1998;54(3):693-702. PubMed PMID: 10326294.

3. Jezek ZaF, F. . Human monkeypox. Monographs in Virology. Karger, editor. Basel1988.

4. Hutin YJ, Williams RJ, Malfait P, Pebody R, Loparev VN, Ropp SL, et al. Outbreak of human monkeypox, Democratic Republic of Congo, 1996 to 1997. Emerging Infectious Diseases. 2001;7(3):434-8. PubMed PMID: 11384521.

5. Rimoin AW, Kisalu N, Kebela-Ilunga B, Mukaba T, Wright LL, Formenty P, et al. Endemic human monkeypox, Democratic Republic of Congo, 2001-2004. Emerging Infectious Diseases. 2007;13(6):934-7. PubMed PMID: 46890148.

6. Rimoin AW, Mulembakani PM, Johnston SC, Lloyd Smith JO, Kisalu NK, Kinkela TL, et al. Major increase in human monkeypox incidence 30 years after smallpox vaccination campaigns cease in the Democratic Republic of Congo. Proceedings of the National Academy of Sciences of the United States of America. 2010;107(37):16262-7. PubMed PMID: 359779207.

7. Nolen LD, Osadebe L, Katomba J, Likofata J, Mukadi D, Monroe B, et al. Extended human-to-human transmission during a monkeypox outbreak in the Democratic Republic of the Congo. Emerging Infectious Diseases. 2016;22(6):1014-21. PubMed PMID: 610425195.

8. McCollum AM, Nakazawa Y, Ndongala GM, Pukuta E, Karhemere S, Lushima RS, et al. Human monkeypox in a conflict region of the democratic republic of the Congo. American Journal of Tropical Medicine and Hygiene. 2013;1):17-8. PubMed PMID: 71311889.

9. Osadebe L, Hughes CM, Shongo Lushima R, Kabamba J, Nguete B, Malekani J, et al. Enhancing case definitions for surveillance of human monkeypox in the Democratic Republic of Congo. PLoS Neglected Tropical Diseases. 2017;11 (9) (no pagination)(e0005857). PubMed PMID: 618542543.

10. Berthet N, Nakoune E, Whist E, Selekon B, Burguire AM, Manuguerra JC, et al. Maculopapular lesions in the Central African Republic. The Lancet. 2011;378(9799):1354. PubMed PMID: 362718602.

11. IFRC. Central African Republic: Monkey Pox Outbreak - Dec 2015. Reliefweb: 2016.

12. Kalthan E, Tenguere J, Ndjapou SG, Koyazengbe TA, Mbomba J, Marada RM, et al. Investigation of an outbreak of monkeypox in an area occupied by armed groups, Central African Republic. Medecine et Maladies Infectieuses. 2018;48(4):263-8. PubMed PMID: 2000564065.

13. WHO. WHO AFRO Outbreaks and Other Emergencies, Week 16: 15 – 21 April 2017 Data as reported by 17:00 21 April 2017. Reliefweb: 2017.

14. Nakoune E, Selekon B, Komoyo GF, Kazanji M, Garba-Ouangole SM, Janssens C, et al. A Nosocomial Outbreak of Human Monkeypox in the Central African Republic. Open Forum Infectious Diseases. 2017;4(4). doi: 10.1093/ofid/ofx168.

15. Learned LA, Reynolds MG, Wassa DW, Li Y, Olson VA, Karem K, et al. Extended interhuman transmission of monkeypox in a hospital community in the Republic of the Congo, 2003. American Journal of Tropical Medicine & Hygiene. 2005;73(2):428-34. PubMed PMID: 16103616.

16. Reynolds MG, Emerson GL, Pukuta E, Karhemere S, Muyembe JJ, Bikindou A, et al. Short report: Detection of human monkeypox in the Republic of the Congo following intensive community education. American Journal of Tropical Medicine and Hygiene. 2013;88(5):982-5. PubMed PMID: 368857566.

17. NCDC. Situation Report: Monkeypox Outbreak in Nigeria. Abuja: 2018.

18. Breman JG, Nakano JH, Coffi E, Godfrey H, Gautun JC. Human poxvirus disease after smallpox eradication. American Journal of Tropical Medicine & Hygiene. 1977;26(2):273-81. PubMed PMID: 192091.

19. WHO. Monkeypox – Cameroon. 2018.

20. ISDR. L. Liberia IDSR Epidemiology Bulletin 2016 Epi Week 51 (December 16 –December 22 ). Reliefweb: 2016.

21. Formenty P, Muntasir MO, Damon I, Chowdhary V, Opoka ML, Monimart C, et al. Human monkeypox outbreak caused by novel virus belonging to Congo Basin clade, Sudan, 2005. Emerging Infectious Diseases. 2010;16(10):1539-45. PubMed PMID: 20875278.

22. Muller G, Meyer A, Gras F, Emmerich P, Kolakowski T, Esposito JJ. Monkeypox virus in liver and spleen of child in Gabon. Lancet. 1988;1(8588):769-70. PubMed PMID: 2895299.

23. Anonymous. Monkeypox, 1991. Gabon. Weekly Epidemiological Record. 1992;67(14):101-2. PubMed PMID: 1314067.

24. Reynolds MG, Yorita KL, Kuehnert MJ, Davidson WB, Huhn GD, Holman RC, et al. Clinical manifestations of human monkeypox influenced by route of infection. Journal of Infectious Diseases. 2006;194(6):773-80. PubMed PMID: 44344124.
