# Supplementary material for: A systematic review of the epidemiology of human monkeypox outbreaks and implications for outbreak strategy
Source: PLoS Negl Trop Dis. 2019 Oct 16;13(10):e0007791. doi: 10.1371/journal.pntd.0007791 (PMC6816577; doi:10.1371/journal.pntd.0007791)
Supplement: S3 Table — (DOCX) [file pntd.0007791.s004.docx]

## S3 Table. Summary of study findings on risk factors for primary introduction of monkeypox.

| **Country** | **Risk Factor** | **OR** | **aOR for vaccination status (95% CI)** | **P Value** | **Number of cases, n (Number of controls, n)** |
| --- | --- | --- | --- | --- | --- |
| DRC [1]* | Vaccinated | 0.1 (0.03, 0.6) | NA | ND | 252 (653) |
|  | Exposure to Gambian rats | ND | 2.6 (1.6, 4.1) | ND |  |
|  | Large terrestrial rodents | ND | 1.8 (1.1, 3.0) | ND |  |
|  | Prosimiens | ND | 1.9 (1.2, 2.8) | ND |  |
|  | Non-human primates | ND | 2.7 (1.4, 4.9) | ND |  |
| DRC [2] | Live in house with a door | 0.07 (0.01, 0.6) | ND | 0.01 | 15 (50) |
|  | Prepared wild animal for consumption | 0.2 (0.09, 0.1) | ND | 0.04 |  |
|  | Ate duiker | 0.15 (0.03, 0.7) | ND | 0.01 |  |
|  | Sleep on floor | 6.1 (1.2, 31.6) | ND | 0.03 |  |
| USA [3] | Vaccinated | 0.3 (0.1, 0.9) | NA | ND | 30 (35)*^†^* |
|  | Touched an infected animal | 3.8 (1.2–11.7) | 4.0 (1.2, 13.4) | ND |  |
|  | Cleaned the cage or touched the bedding of an infected animal | 5.3 (1.5, 18.9) | 5.3 (1.4, 20.7) | ND |  |
|  | Received scratch from infected animal | 5.6 (1.1, 28.6) | 3.9 (0.7, 21.1) | ND |  |
|  | Daily indirect or direct exposure to an ill animal was significantly associated with MPX developing | 3.8 (1.2, 11.7) | 4.0 (1.2, 13.4) | ND |  |
|  | Having come within 6 feet but not touched by an infected animal | 2.0 (0.6, 6.2) | 2.0 (0.6, 6.5) | ND |  |

NA = Not applicable ND = Not Described *Conference abstract, no methodology provided ^†^Three controls were found to have elevated levels of IgM to orthopoxvirus.

1. Hoff N, Mulembakani PM, Johnston SC, Kisalu NK, Muyembe JJ, Hensley LE, et al. Risk factors associated with human monkeypox in the democratic republic of Congo. American Journal of Tropical Medicine and Hygiene. 2014;1):199-200. PubMed PMID: 71691886.

2. Nolen LD, Osadebe L, Katomba J, Likofata J, Mukadi D, Monroe B, et al. Introduction of Monkeypox into a Community and Household: Risk Factors and Zoonotic Reservoirs in the Democratic Republic of the Congo. American Journal of Tropical Medicine & Hygiene. 2015;93(2):410-5. PubMed PMID: 26013374.

3. Reynolds MG, Davidson WB, Curns AT, Conover CS, Huhn G, Davis JP, et al. Spectrum of infection and risk factors for human monkeypox, United States, 2003. Emerging Infectious Diseases. 2007;13(9):1332-9. PubMed PMID: 47510294.
