# Supplementary material for: A systematic review of the epidemiology of human monkeypox outbreaks and implications for outbreak strategy
Source: PLoS Negl Trop Dis. 2019 Oct 16;13(10):e0007791. doi: 10.1371/journal.pntd.0007791 (PMC6816577; doi:10.1371/journal.pntd.0007791)
Supplement: S4 Table — (DOCX) [file pntd.0007791.s005.docx]

## S4 Table. Case Definition Summary.

| Country | Authors, context and purpose | Suspect Case Definition(s) | Components |
| --- | --- | --- | --- |
| DRC | MOH 2011 [1, 2], monkeypox surveillance. ‘A’ intended for sensitive detection by surveillance system, ‘B’ for discriminating monkeypox over other illness namely VZV, at outbreak level | *A: ‘Any person appearing with a sudden onset of high fever, followed a few days later by a vesicular–pustular eruption presenting predominantly on the face, palms of the hands, and soles of the feet; or the presence of at least five smallpox type scabs’*  B ‘*Vesicular or pustular eruption with deep-seated, firm pustules and at least one of the following symptoms: fever preceding the eruption, lymphadenopathy (inguinal, axillary, or cervical), and/or pustules or crusts on the palms of the hands or soles of the feet.’ (known to have been a MOH definition used for a 2009-14 cohort)* | A: Febrile prodrome  PLUS  Vesicular or pustular rash OR scars on palm/soles/face OR 5+ scars  B: Vesicular pustular rash PLUS LEAST ONE of: febrile prodrome AND/OR lymphadenopathy OR palm/sole rash distribution |
|  | Case definition evaluation study plus summative symptom criteria analysis [1] | See above. First definition named A, second named B.  A receiver operating characteristic ROC analysis was also conducted using a requisite ‘febrile prodrome’ and up to 12 signs/symptom criteria (nausea, lymphadenopathy, fatigue, conjunctivitis, bedridden, lesions on genitals, lesions of the same size, lesions on arms, legs, palms or soles) | See above. First definition named A, second named B.  ROC: Combinations with febrile prodrome starting point |
|  | Aketi HZ Outbreak investigation level: active case finding [2] | MOH definition A plus use of a symptom score | See MOH |
|  | Outbreak investigation level [3] | *A person with a history of high fever and a vesicular-pustular rash and with at least one of the following three characteristics: 1) rash on the palms and soles, 2) lymphadenopathy, and/or 3) fever preceding rash. (July 1^st^-December 8^th^ 2013)* | Fever PLUS vesicular pustular rash PLUS AT LEAST ONE: palm/sole distribution AND/OR lymphadenopathy AND/OR febrile prodrome |
|  | Used by McMullen et al 2015 [4], Hoff et al 2014 [5], Rimoin et al 2010 [6]: Passive surveillance purposes. Retrospective analysis studies. | *Fever* ≥ *38*°C *and vesiculopustular rash* | Fever PLUS Rash |
|  | Case definition evaluation (abstract) [7] | See MOH definition A | See MOH A |
| ROC | Used for retrospective analysis of presentations in medical records [8] | V*esicular pustular eruption characterized by hard and deep pustules not explained by any other disease, and a febrile prodrome, or lymphadenopathy (axillary, auricular, cervical), or lesions on the palms of the hands and soles of the feet* | Vesicular pustular rash AND febrile prodrome OR lymphadenopathy OR palms/soles |
| Nigeria | Active surveillance during outbreak investigations 2017-18. Assuming NCDC definition was used [9, 10] | *Any person presenting with a history of sudden onset of fever, followed by a vesiculopustular rash occurring mostly on the face, palms and soles of feet.* | Febrile prodrome PLUS rash mostly on face/palms/soles |
| USA | Created by CDC with aim to detect all cases in persons exposed to imported ill animals in 2003 outbreak [11-13] Adapted version used in ROC [14] | *Meets one of the epidemiologic criteria*  *AND*  *Fever or unexplained rash AND two or more other signs or symptoms with onset of first sign or symptom <21 days after last exposure meeting epidemiologic criteria*  *Epidemiologic criteria:*  *Exposure^1^ to an exotic or wild mammalian pet^2^ obtained on or after April 15, 2003, with clinical signs of illness (e.g., conjunctivitis, respiratory symptoms, and/or rash)*  *Exposure^1^ to an exotic or wild mammalian pet^2^ with or without clinical signs of illness that has been in contact with either a mammalian pet^3^ or a human with monkeypox*  *Exposure^4^ to a suspect, probable, or confirmed human case of monkeypox*  *Exclusion Criteria:*  *A case may be excluded as a suspect or probable monkeypox case if:*  *An alternative diagnosis can fully explain the illness^5^*  *OR*  *The case was reported on the basis of primary or secondary exposure to an exotic or wild mammalian pet or a human subsequently determined not to have monkeypox, provided other possible epidemiologic exposure criteria are not present*  *OR*  *A case without a rash does not develop a rash within 10 days of onset of clinical symptoms consistent with monkeypox^6^.*  *The case is determined to be negative for non-variola generic orthopoxvirus by polymerase chain reaction testing of a well sampled rash lesion by the approved Laboratory Response Network (LRN) protocol.* | Fever OR rash plus any other symptom PLUS exposure features specific to outbreak source |
| CAR | Outbreak investigation. Active surveillance at village level presumed to be at time of outbreak. [15] | *Any individual living in the district of Alindao as of July 15, 2016 and presenting with fever and vesicular or pustular skin rash.* | Fever PLUS rash |
| Southern Sudan | Retrospective outbreak investigation conducted one- month post-outbreak. [16] | *Any person from the outbreak zone who sought treatment during September 2005 - January 2006 for fever (>37.5C) and vesicular crusty rash.* | Fever PLUS rash |

VP = vesiculopustular HZ = Health Zone ‘1) Includes living in a household, petting or handling, or visiting a pet holding facility (e.g., pet store, veterinary clinic, pet distributor 2) Includes prairie dogs, Gambian giant rats, and rope squirrels. Exposure to other exotic or non-exotic mammalian pets will be considered on a case-by-case basis; assessment should include the likelihood of contact with a mammal with monkeypox and the compatibility of clinical illness with monkeypox 3) Includes living in a household, or originating from the same pet holding facility as another animal with monkeypox 4) Includes skin-to-skin or face-to-face contact 5) Factors that might be considered in assigning alternate diagnoses include the strength of the epidemiologic exposure criteria for monkeypox, the specificity of the diagnostic test, and the compatibility of the clinical presentation and course of illness for the alternative diagnosis. 6) If possible, obtain convalescent-phase serum specimen from these patients. See specimen collection guidelines for details on collecting serum for convalescence evaluation’.

1. Osadebe L, Hughes CM, Shongo Lushima R, Kabamba J, Nguete B, Malekani J, et al. Enhancing case definitions for surveillance of human monkeypox in the Democratic Republic of Congo. PLoS Neglected Tropical Diseases. 2017;11 (9) (no pagination)(e0005857). PubMed PMID: 618542543.

2. Laudisoit A. Bushmeat and Monkeypox: Yahuma Health Zone – Aketi Health Zone - Bombongolo Health Area. Kisangani, DRC.: CIFOR, Université de Kisangani, DRC., 2016.

3. Nolen LD, Osadebe L, Katomba J, Likofata J, Mukadi D, Monroe B, et al. Extended human-to-human transmission during a monkeypox outbreak in the Democratic Republic of the Congo. Emerging Infectious Diseases. 2016;22(6):1014-21. PubMed PMID: 610425195.

4. McMullen CL, Mulembekani P, Hoff NA, Doshi RH, Mukadi P, Shongo R, et al. Human monkeypox transmission dynamics thirty years after smallpox eradication in the Sankuru district, democratic republic of Congo. American Journal of Tropical Medicine and Hygiene. 2015;93 (4 Supplement):341. PubMed PMID: 613369164.

5. Hoff N, Ilunga BK, Shongo R, Muyembe JJ, Mossoko M, Okitolonda E, et al. Human monkeypox disease surveillance and time trends in The Democratic Republic of Congo, 2001-2013. American Journal of Tropical Medicine and Hygiene. 2014;1):339. PubMed PMID: 71692341.

6. Rimoin AW, Mulembakani PM, Johnston SC, Lloyd Smith JO, Kisalu NK, Kinkela TL, et al. Major increase in human monkeypox incidence 30 years after smallpox vaccination campaigns cease in the Democratic Republic of Congo. Proceedings of the National Academy of Sciences of the United States of America. 2010;107(37):16262-7. PubMed PMID: 359779207.

7. McCollum AM, Balilo MP, Pukuta E, Muyembe JJ, Damon IK, Reynolds MG. Towards enhanced surveillance for monkeypox: Application of a robust clinical case definition. American Journal of Tropical Medicine and Hygiene. 2010;1):122. PubMed PMID: 70442417.

8. Reynolds MG, Emerson GL, Pukuta E, Karhemere S, Muyembe JJ, Bikindou A, et al. Short report: Detection of human monkeypox in the Republic of the Congo following intensive community education. American Journal of Tropical Medicine and Hygiene. 2013;88(5):982-5. PubMed PMID: 368857566.

9. Yinka-Ogunleye A, Aruna O, Ogoina D, Aworabhi N, Eteng W, Badaru S, et al. Reemergence of human monkeypox in Nigeria, 2017. Emerging Infectious Diseases. 2018;24(6):1149-51. PubMed PMID: 622264241.

10. NCDC. Situation Report: Monkeypox Outbreak in Nigeria. Abuja: 2018.

11. Reynolds MG, Cono J, Curns A, Holman RC, Likos A, Regnery R, et al. Human monkeypox. The Lancet Infectious Diseases. 2004;4(10):604-5; discussion 5. PubMed PMID: 15451482.

12. Sejvar JJ, Chowdary Y, Schomogyi M, Stevens J, Patel J, Karem K, et al. Human monkeypox infection: A family cluster in the Midwestern United States. Journal of Infectious Diseases. 2004;190(10):1833-40. PubMed PMID: 39487830.

13. Anderson MG, Frenkel LD, Homann S, Guffey J. A case of severe monkeypox virus disease in an American child: Emerging infections and changing professional values. Pediatric Infectious Disease Journal. 2003;22(12):1093-6. PubMed PMID: 37543410.

14. Learned LA, Reynolds MG, Wassa DW, Li Y, Olson VA, Karem K, et al. Extended interhuman transmission of monkeypox in a hospital community in the Republic of the Congo, 2003. American Journal of Tropical Medicine & Hygiene. 2005;73(2):428-34. PubMed PMID: 16103616.

15. Kalthan E, Tenguere J, Ndjapou SG, Koyazengbe TA, Mbomba J, Marada RM, et al. Investigation of an outbreak of monkeypox in an area occupied by armed groups, Central African Republic. Medecine et Maladies Infectieuses. 2018;48(4):263-8. PubMed PMID: 2000564065.

16. Formenty P, Muntasir MO, Damon I, Chowdhary V, Opoka ML, Monimart C, et al. Human monkeypox outbreak caused by novel virus belonging to Congo Basin clade, Sudan, 2005. Emerging Infectious Diseases. 2010;16(10):1539-45. PubMed PMID: 20875278.
