# Supplementary material for: A systematic review of the epidemiology of human monkeypox outbreaks and implications for outbreak strategy
Source: PLoS Negl Trop Dis. 2019 Oct 16;13(10):e0007791. doi: 10.1371/journal.pntd.0007791 (PMC6816577; doi:10.1371/journal.pntd.0007791)
Supplement: S5 Table — (DOCX) [file pntd.0007791.s006.docx]

## S5 Table. Secondary Attack Rate Table.

| **Measure** | **Study** | **Year(s)** | **Secondary cases** | **Secondary Contacts** | **SAR** | **Lower 95% CI** | **Upper 95% CI** |
| --- | --- | --- | --- | --- | --- | --- | --- |
| **Overall SAR in Household Contacts** | Jezek [1] | 1980-5 | 5 | 190 | 2.6 | 0.4 | 4.9 |
|  | Jezek [2] | 1981-6 | 53 | 1420 | 3.7 | 2.8 | 4.7 |
|  | Fine [3] | 1980-4 | 36 | 834 | 4.3 | 2.9 | 5.7 |
|  | CDC [4] | 1996-7 | 20 | 240 | 8.3 | 4.8 | 11.8 |
|  | McMullen_a_ [5] | 2005-7 | 88 | 1214 | 7.3 | 5.8 | 8.7 |
|  | McMullen_b_ [5] | 2005-7 | 140 | 1509 | 9.3 | 7.8 | 10.7 |
|  | McCollum [6] | 2012 | 0 | 7 | 0.0 | 0.0 | 0.4 |
| **SAR in Unvaccinated Household Contacts** | Jezek | 1980-5 | 3 | 25 | 12.0 | 0.0 | 24.7 |
|  | Jezek | 1981-6 | 40 | 431 | 9.3 | 6.5 | 12.0 |
|  | Fine | 1980-4 | 26 | 236 | 11.0 | 9.0 | 13.1 |
|  | McCollum | 2012 | 0 | 2 | 0.0 | 0.0 | 0.8 |
| **SAR in Vaccinated Household Contacts** | Jezek | 1980-5 | 2 | 165 | 1.2 | 0.4 | 2.1 |
|  | Jezek | 1981-6 | 13 | 989 | 1.3 | 0.6 | 2.0 |
|  | Fine | 1980-4 | 10 | 598 | 1.7 | 0.6 | 2.7 |
|  | McCollum | 2012 | 0 | 5 | 0.0 | 0.0 | 0.5 |
| **Overall SAR in Non-Household Contacts** | Jezek | 1981-6 | 16 | 858 | 1.9 | 1.0 | 2.8 |
|  | Fine | 1980-4 | 11 | 739 | 1.5 | 0.6 | 2.4 |
|  | McMullen_a_ | 2005-7 | 10 | 211 | 4.7 | 1.9 | 7.6 |
|  | McMullen_b_ | 2005-7 | 25 | 277 | 9.0 | 5.7 | 12.4 |
|  | McCollum | 2012 | 0 | 23 | 0.0 | 0.0 | 0.2 |
| **SAR in Unvaccinated Non-household Contacts** | Jezek | 1981-6 | 14 | 292 | 4.8 | 2.3 | 7.3 |
|  | Fine | 1980-4 | 9 | 238 | 3.8 | 1.4 | 6.2 |
|  | McCollum | 2012 | 0 | 14 | 0.0 | 0.0 | 0.2 |
| **SAR in Vaccinated Non-Household Contacts** | Jezek | 1981-6 | 2 | 566 | 0.4 | 0.0 | 0.9 |
|  | Fine | 1980-4 | 2 | 501 | 0.4 | 0.0 | 1.0 |
|  | McCollum | 2012 | 0 | 9 | 0.0 | 0.0 | 0.3 |

1. Jezek ZaF, F. . Human monkeypox. Monographs in Virology. Karger, editor. Basel1988.

2. Jezek Z, Grab B, Szczeniowski MV, Paluku KM, Mutombo M. Human monkeypox: secondary attack rates. Bulletin of the World Health Organization. 1988;66(4):465-70. PubMed PMID: 18222133.

3. Fine PE, Jezek Z, Grab B, Dixon H. The transmission potential of monkeypox virus in human populations. International Journal of Epidemiology. 1988;17(3):643-50. PubMed PMID: 2850277.

4. Prevention. CfDCa. Human monkeypox -- Kasai Oriental, Democratic Republic of Congo, February 1996-October 1997. MMWR - Morbidity & Mortality Weekly Report. 1997;46(49):1168-71. PubMed PMID: 9408046.

5. McMullen CL, Mulembekani P, Hoff NA, Doshi RH, Mukadi P, Shongo R, et al. Human monkeypox transmission dynamics thirty years after smallpox eradication in the Sankuru district, democratic republic of Congo. American Journal of Tropical Medicine and Hygiene. 2015;93 (4 Supplement):341. PubMed PMID: 613369164.

6. McCollum AM, Nakazawa Y, Ndongala GM, Pukuta E, Karhemere S, Lushima RS, et al. Human monkeypox in a conflict region of the democratic republic of the Congo. American Journal of Tropical Medicine and Hygiene. 2013;1):17-8. PubMed PMID: 71311889.
