# Supplementary material for: A systematic review of the epidemiology of human monkeypox outbreaks and implications for outbreak strategy
Source: PLoS Negl Trop Dis. 2019 Oct 16;13(10):e0007791. doi: 10.1371/journal.pntd.0007791 (PMC6816577; doi:10.1371/journal.pntd.0007791)
Supplement: S6 Table — (DOCX) [file pntd.0007791.s007.docx]

## S6 Table. Suspected, confirmed and fatal monkeypox cases by country and year.

Each box denotes number of cases, n, number of confirmed cases (n), and number of deaths, n, and/or CFR (%). Where suspect cases were tested and found positive for another disease, these were subtracted from the suspect case total. CFR calculated as number of deaths out of total suspect cases. Grey denotes a period without reported outbreaks. Green denotes locations affected by CB clade. Orange denotes areas affected by WA clade. Table does not include the USA 2003 imported outbreak 06/03-06/2003: 47 (37) 0%

| Country | DRC (Zaire 1971-97)* | South Sudan (Sudan pre-2011) | Gabon | Cameroon | CAR | ROC | Sierra Leone | Cote d’Ivoire | Nigeria | Liberia |
| --- | --- | --- | --- | --- | --- | --- | --- | --- | --- | --- |
| 1970-1975 | 1970: 1 (1) 100% [1]  1971: 0  1972: 5  1973: 3  1974: 1 |  |  |  |  |  | 1970-71:  1 (1) 0% [2] | 10/1971:  1 (U) 0% [2] | 1971: 2 (1) 0% [2] | 1970-71:  4 (1) 0% [2] |
| 1975-1980 | 1975: 3  1976: 5  1977: 6  1978: 12  1979: 8 |  |  | 1979: 2 (U) U% [3] |  |  |  |  | 1978: 1 (1) 0% [2] |  |
| 1980-1985 | 1980: 4  1981: 7  1982: 40  1983: 84  1984: 86 |  | 1987: Lambarene: 1 (1) 100%^‖ ‖^ [4] |  | 1984: 6 (U) U% [3] |  |  | 1981: 1 (U) U% [3] |  |  |
| 1985-1990 | 1985: 62  1986: 59 [3]  1981-86: 338 (U) 9.8% [3] |  |  | 1989: 1( 1) 0% [5] |  |  |  |  |  |  |
| 1990-1995 |  |  | 01/1991-06/1991: Region between Lamberene and N’Djole: 9 (5) 0% [6] |  |  |  |  |  |  |  |
| 1995-2000 | 02/1996-02/1997: Katako-Kombe HZ: 92 (11) 3.3%^‡‡^ [7]  02/1996 – 10/1997: Kasai Oriental: 511 (U) (1.5%) ^§§^ [8]  03/1997 - 05/1997: Katako-Kombe n=112, Lodja Nord n=58, Sud HZ n=11: 170 (U) 0% (Weekly Epidemiological Record) [9] |  |  |  |  |  |  |  |  |  |
| 2000** -2005 | 02/2001-08/2001: Equateur Province: 23 (9) 21.7% [10]  2001: 388 (U) n=13 (3.4%)  2002: 881 (1.6%)  2003: 755 (2.1%)  2004: 1024 (2.8%) |  |  |  | 14/08/2001: Pimu CAR/DRC border: 8 cases in a family (U) 25% [11] ^¶¶^ | 15/04/03 – 23/06/2003: Likouala department:  12 (3) 8.3%†  [12] |  |  |  |  |
| 2005-2010 | 2005: 1708 (1.5%)  2006: 783 (2.6%)  2005-7: Sankuru District: 1407 (703) U% [13]  2007: 970 (1.1%)  2008: 1599 (4.2%)  2009: 1919 (1.4%) | 10/2005: Unity State:  37 (10) 0% [14] |  |  |  | 2007: Likouala department: 62 - 150+ (U) U%‡ (IRIN, Reliefweb) [15] |  |  |  |  |
| 2010-2015 | 2010: 2322 (1.1%)  2011: 2208 (0.7%)  2012: 2629 (1.3%)  2013: 2460 (1.5%)†† (IDSR data) [16]  2013: Bokungu HZ: 99 (50) 10 (10.1%) [17] ^¶^ |  |  |  | 06/2010: Deep forest, Southern CAR, 480km from DRC border: 2 (2) 0%^§^ [11]  30/04/2012: Batangafo: 2 (U) U% [11] | 04/10-11/2010: Likouala department:  11 (2) 9.1% [18] | 2014: Bo District: 1 (U) U% [19] |  |  |  |
| 2015-present | 1/1/2016 -1/3/2016: Ateki HZ: 155 (7) 11 (7.1%) [20]  01/01/2018 - 08/07/2018: IDSR: 2995 (U) 1.2% [21] |  |  | 30/04/2018: Njikwa Health District: 6 (1) 0% [22] | 30/01/2015: Bria: 3 (U) n=1 33%  04/12/2015 - 02/2016: Bakouma and Bangassou subprefectures, Mbomou province: 10 (3) 20% [23]  04/12/2015-28/09/2016: Additional 3 cases reported^‖^ (IFRC) [24]  04/09/16-07/10/2016: Haute-Kotto health district: 26 (3) 3.8%*** [25]  08/2016-10/2016: Alindao-Mingala Health District: 26 (3) 7.7% [26]  2017: 01/17-03/17: Ouango district, DRC border: 47 (5) U% [19]  12/04/2017: M’baïki district: 2 (1) 0% [19]  17/03/2018: Bambari, Ippy sub-district. 9 (6) 0% [27]  29/03/17: Bangassou, sub-district Rafai: 15 (3) 6.7% [24]  30/06/2018: Mbaïki, Bangandou sub-district: 5 (2) 0% [21] | 18/01/17 – 15/10/17: Likouala department: 88 (7) 6.8%  [28] | 14/03/2017: Pujehun district:  1 (1) 0% [19] |  | 2017-18: 24 States:  228 (89) 2.6% [29] | 12/2016  1 (U) U% [30]  07/2017: 1 (U) U% (Liberia IDSR) [31]  26/11/ 2017: 1 (0) U% (Liberia IDSR) [32] |

HZ = Health Zone U = Unknown *Outbreaks and case counts provided for DRC where found: monkeypox is endemic and over 1000 suspect cases are recorded each year. †n=1 death however cause of death unclear as post-operative peritoneal infection after exploratory surgery, as well as acute MPX. N=1 experienced viral conjunctivitis 6 weeks post-MPX with extensive corneal damage. N=1 infant did not regain full health and died 6 months later with reported haemolytic anaemia however details unclear. ‡IRIN press report. §Assumed no fatalities. ‖ IFRC values used. ¶ 104 suspect cases reported by Nolen et al 2016. 5 VZV positive cases subtracted from total, 5 negatives for MPXV and VZV included in total. **Monkeypox became reportable to the IDSR in DRC in 2000. ††IDSR data, accessed via Hoff (2014) at height of reporting, only 26.5% of HZ were reporting monkeypox cases. ‡‡Most up-to-date corrected values used for this outbreak. §§Out of 344 cases identified in Katako-Kombe HZ. ‖‖The 9-month old girl was co-infected with Plasmodium falciparum. ¶¶Referenced in Berthet et al, supplementary data in Nakoune et al. *** Death of index case reported, other unreported.

1. Ladnyj ID, Ziegler P, Kima E. A human infection caused by monkeypox virus in Basankusu Territory, Democratic Republic of the Congo. Bulletin of the World Health Organization. 1972;46(5):593-7. PubMed PMID: 293103001.

2. Breman JG, Kalisa R, Steniowski MV, Zanotto E, Gromyko AI, Arita I. Human monkeypox, 1970-79. Bulletin of the World Health Organization. 1980;58(2):165-82. PubMed PMID: 6249508.

3. Jezek ZaF, F. . Human monkeypox. Monographs in Virology. Karger, editor. Basel1988.

4. Muller G, Meyer A, Gras F, Emmerich P, Kolakowski T, Esposito JJ. Monkeypox virus in liver and spleen of child in Gabon. Lancet. 1988;1(8588):769-70. PubMed PMID: 2895299.

5. Tchokoteu PF, Kago I, Tetanye E, Ndoumbe P, Pignon D, Mbede J. [Variola or a severe case of varicella? A case of human variola due to monkeypox virus in a child from the Cameroon]. Ann Soc Belg Med Trop. 1991;71(2):123-8. Epub 1991/06/01. PubMed PMID: 1656900.

6. Record. WE. Monkeypox, 1991. Gabon. Weekly Epidemiological Record. 1992;67(14):101-2. PubMed PMID: 1314067.

7. Record. WE. Monkeypox in the Democratic Republic of the Congo (former Zaire). Weekly Epidemiological Record. 1997;72(34):258. PubMed PMID: 9283251.

8. Mukinda VB, Mwema G, Kilundu M, Heymann DL, Khan AS, Esposito JJ. Re-emergence of human monkeypox in Zaire in 1996. Monkeypox Epidemiologic Working Group. Lancet. 1997;349(9063):1449-50. PubMed PMID: 9164323.

9. Prevention. CfDCa. Human monkeypox -- Kasai Oriental, Democratic Republic of Congo, February 1996-October 1997. MMWR - Morbidity & Mortality Weekly Report. 1997;46(49):1168-71. PubMed PMID: 9408046.

10. Meyer H, Perrichot M, Stemmler M, Emmerich P, Schmitz H, Varaine F, et al. Outbreaks of disease suspected of being due to human monkeypox virus infection in the Democratic Republic of Congo in 2001. Journal of Clinical Microbiology. 2002;40(8):2919-21. PubMed PMID: 12149352.

11. Berthet N, Nakoune E, Whist E, Selekon B, Burguire AM, Manuguerra JC, et al. Maculopapular lesions in the Central African Republic. The Lancet. 2011;378(9799):1354. PubMed PMID: 362718602.

12. Learned LA, Reynolds MG, Wassa DW, Li Y, Olson VA, Karem K, et al. Extended interhuman transmission of monkeypox in a hospital community in the Republic of the Congo, 2003. American Journal of Tropical Medicine & Hygiene. 2005;73(2):428-34. PubMed PMID: 16103616.

13. McMullen CL, Mulembekani P, Hoff NA, Doshi RH, Mukadi P, Shongo R, et al. Human monkeypox transmission dynamics thirty years after smallpox eradication in the Sankuru district, democratic republic of Congo. American Journal of Tropical Medicine and Hygiene. 2015;93 (4 Supplement):341. PubMed PMID: 613369164.

14. Formenty P, Muntasir MO, Damon I, Chowdhary V, Opoka ML, Monimart C, et al. Human monkeypox outbreak caused by novel virus belonging to Congo Basin clade, Sudan, 2005. Emerging Infectious Diseases. 2010;16(10):1539-45. PubMed PMID: 20875278.

15. Congo: Monkeypox infects 60 in North [Internet]. Reliefweb: Reliefweb; 2007; 25/09/2007

16. Hoff NA, Kebela-Ilunga B, Eckhoff P, Mukadi P, Mossoko M, Muyembe-Tamfum JJ, et al. A descriptive and quantitative analysis of potential underestimation of human monkeypox cases in the passive surveillance system in the democratic republic of congo. American Journal of Tropical Medicine and Hygiene. 2015;93 (4 Supplement):242. PubMed PMID: 613370358.

17. Nolen LD, Osadebe L, Katomba J, Likofata J, Mukadi D, Monroe B, et al. Extended human-to-human transmission during a monkeypox outbreak in the Democratic Republic of the Congo. Emerging Infectious Diseases. 2016;22(6):1014-21. PubMed PMID: 610425195.

18. Reynolds MG, Emerson GL, Pukuta E, Karhemere S, Muyembe JJ, Bikindou A, et al. Short report: Detection of human monkeypox in the Republic of the Congo following intensive community education. American Journal of Tropical Medicine and Hygiene. 2013;88(5):982-5. PubMed PMID: 368857566.

19. WHO. WHO AFRO Outbreaks and Other Emergencies, Week 16: 15 – 21 April 2017 Data as reported by 17:00 21 April 2017. Reliefweb: 2017.

20. Laudisoit A. Bushmeat and Monkeypox: Yahuma Health Zone – Aketi Health Zone - Bombongolo Health Area. Kisangani, DRC.: CIFOR, Université de Kisangani, DRC., 2016.

21. WHO. WHO AFRO Outbreaks and Other Emergencies, Week 30: 21 -27 July 2018 (Data as reported by 17:00; 27 July 2018). . Reliefweb: 2018.

22. WHO. Monkeypox – Cameroon. 2018.

23. Nakoune E, Selekon B, Komoyo GF, Kazanji M, Garba-Ouangole SM, Janssens C, et al. A Nosocomial Outbreak of Human Monkeypox in the Central African Republic. Open Forum Infectious Diseases. 2017;4(4). doi: 10.1093/ofid/ofx168.

24. IFRC. Central African Republic: Monkey Pox Outbreak - Dec 2015. Reliefweb: 2016.

25. WHO. Monkeypox in Central African Republic. Reliefweb: 2016.

26. Kalthan E, Tenguere J, Ndjapou SG, Koyazengbe TA, Mbomba J, Marada RM, et al. Investigation of an outbreak of monkeypox in an area occupied by armed groups, Central African Republic. Medecine et Maladies Infectieuses. 2018;48(4):263-8. PubMed PMID: 2000564065.

27. WHO. WHO AFRO Outbreaks and Other Emergencies, Week 13: 25 – 31 March 2017 Data as reported by 17:00 31 March. . Reliefweb: 2017.

28. WHO. WHO AFRO Outbreaks and Other Emergencies, Week 48: 25 November - 01 December 2017 (Data as reported by 17:00; 1 December 2017). Reliefweb: 2017.

29. NCDC. Situation Report: Monkeypox Outbreak in Nigeria. Abuja: 2018.

30. ISDR. L. Liberia IDSR Epidemiology Bulletin 2016 Epi Week 51 (December 16 –December 22 ). Reliefweb: 2016.

31. ISDR. L. Liberia IDSR Epidemiology Bulletin 2017 Epi Week 19 (May 8 –14, 2017). Reliefweb: 2017.

32. ISDR. L. Liberia IDSR Epidemiology Bulletin 2017 Epi Week 49 (December 4 –December 10). Reliefweb: 2017.
